# Application of Virtual Reality-Assisted Exergaming on the Rehabilitation of Children with Cerebral Palsy: A Systematic Review and Meta-Analysis

**DOI:** 10.3390/jcm12227091

**Published:** 2023-11-14

**Authors:** Muhammad Abubaker Tobaiqi, Emad Ali Albadawi, Hammad Ali Fadlalmola, Muayad Saud Albadrani

**Affiliations:** 1 Department of Family and Community Medicine, College of Medicine, Taibah University, Al-Madinah Al-Munawara 42353, Saudi Arabia; 2 Department of Anatomy, College of Medicine, Taibah University, Al-Madinah Al-Munawara 42353, Saudi Arabia; dr-albadawi@hotmail.com; 3 Department of Community Health Nursing, Nursing College, Taibah University, Al-Madinah Al-Munawara 42353, Saudi Arabia; hazzminno345@gmail.com

**Keywords:** cerebral palsy, exergaming, rehabilitation, virtual reality, systematic review, meta-analysis

## Abstract

Background: Rehabilitation programs for children with cerebral palsy (CP) aim to improve their motor and cognitive skills through repeated and progressively challenging exercises. However, these exercises can be tedious and demotivating, which can affect the effectiveness and feasibility of the programs. To overcome this problem, virtual reality VR-assisted exergaming has emerged as a novel modality of physiotherapy that combines fun and motivation with physical activity. VR exergaming allows children with CP to perform complex movements in a secure and immersive environment, where they can interact with virtual objects and scenarios. This enhances their active engagement and learning, as well as their self-confidence and enjoyment. We aim to provide a comprehensive overview of the current state of research on VR exergaming for CP rehabilitation. The specific objectives are: To identify and describe the existing studies that have investigated the effects of VR exergaming on motor function and participation outcomes in children with CP. In addition, we aim to identify and discuss the main gaps, challenges, and limitations in the current research on VR exergaming for CP rehabilitation. Finally, we aim to provide recommendations and suggestions for future research and practice in this field. Methods: In June 2023, we conducted a systematic search on Scopus, Web of Science, PubMed, Cochrane, and Embase for randomized trials and cohort studies that applied VR-assisted exergaming to rehabilitating patients with CP. The inclusion criteria encompassed the following: (1) Randomized controlled trials (RCTs) and cohort studies involving the rehabilitation of children with CP; (2) the application of VR-based exergaming on the rehabilitation; (3) in comparison with conventional rehabilitation/usual care. The quality of the selected RCTs was evaluated using Cochrane’s tool for risk of bias assessment bias includes. Whereas the quality of cohort studies was assessed using the National Institutes of Health (NIH) tool. Results: The systematic search of databases retrieved a total of 2576 studies. After removing 863 duplicates, 1713 studies underwent title and abstract screening, and 68 studies were then selected as eligible for full-text screening. Finally, 45 studies were involved in this review (n = 1580), and 24 of those were included in the quantitative analysis. The majority of the included RCTs had a low risk of bias regarding study reporting, participants’ attrition, and generating a random sequence. Nearly half of the RCTs ensured good blinding of outcomes assessors. However, almost all the RCTs were unclear regarding the blinding of the participants and the study personnel. The 2020 retrospective cohort study conducted at Samsung Changwon Hospital, investigating the effects of virtual reality-based rehabilitation on upper extremity function in children with cerebral palsy, demonstrated fair quality in its methodology and findings. VR-assisted exergaming was more effective than conventional physiotherapy in improving the Gross Motor Function Measurement (GMFM)-88 score (MD = 0.81; 95% CI [0.15, 1.47], *p*-value = 0.02) and the GMFM walking and standing dimensions (MD = 1.45; 95% CI [0.48, 2.24], *p*-value = 0.003 and MD = 3.15; 95% CI [0.87, 5.42], *p*-value = 0.007), respectively. The mobility and cognitive domains of the Pediatric Evaluation of Disability Inventory score (MD = 1.32; 95% CI [1.11, 1.52], *p*-value < 0.001) and (MD = 0.81; 95% CI [0.50, 1.13], *p*-value < 0.0001) were also improved. The Canadian Occupational Performance Measure performance domain (MD = 1.30; 95% CI [1.04, 1.56], *p*-value < 0.001), the WeeFunctional Independence Measure total score (MD = 6.67; 95% CI [6.36, 6.99], *p*-value < 0.0001), and the Melbourne Assessment of Unilateral Upper Limb Function-2 score (*p*-value < 0.001) improved as well. This new intervention is similarly beneficial as conventional therapy in improving other efficacy measures. Conclusions: Our findings suggest that VR-assisted exergaming may have some advantages over conventional rehabilitation in improving CP children’s functioning and performance in daily life activities, upper and lower limb mobility, and cognition. VR-assisted exergaming seems to be as effective as conventional physiotherapy in the other studied function measures. With its potential efficacy, better feasibility, no reported side effects, and entertaining experience, VR-assisted exergaming may be a viable complementary approach to conventional physiotherapy in rehabilitating children with CP.

## 1. Introduction

Cerebral palsy (CP) is a permanent neurodevelopmental disorder [1]. The term covers a group of conditions that result from a non-progressive lesion affecting the developing brain before, during, or after birth. This lesion causes motor impairment that affects mainly patients’ movement and posture development [2,3,4]. Motor impairment might also be accompanied by sensory, cognitive, or communication deficits or even behavioral abnormalities. Epilepsy and learning difficulties usually coincide with CP as well [5,6,7]. Cerebral palsy prevalence has been increasing over the past years, and it is considered the most common cause of childhood neurological disability worldwide [2,8].

Body mass growth is normally accompanied by an increase in muscle mass, however, this increase in muscle mass is defective in children with CP [9]. Children with CP have motor abnormalities, such as abnormal tone, weakness, and control that affect balance, posture, and coordination, and cause contractures and deformities. These symptoms impair their daily life activities [5,10,11,12,13].

Children with CP need rehabilitation to increase muscle mass and motor function, which can boost their independence and daily life performance. Effective neurorehabilitation requires repeated and varied tasks with increasing difficulty, which can help the brain form new muscle synergies for specific goals [7,14,15,16]. However, for several reasons, the difficulty comes in sustaining physiotherapy throughout the individual’s whole life span.

With the recent advances in technology, rehabilitation through virtual reality (VR)-assisted exergaming was introduced as a complementary approach for CP patients [17,18,19,20,21]. Virtual reality technology provides an interactive computerized simulation of a real-world environment. The technology reacts with the users’ real-time movement using three-dimensional sensors, which are devices that can measure the strength or direction of a magnetic or electric field in three dimensions, i.e., along the x-, y-, and z-axes. They can be used for various applications, such as position detection, motion tracking, gesture recognition, and robotics. The users move and engage in the video game with all their senses (touch, hearing, and vision), and the sensors give direct encouraging feedback on their performance [22,23,24,25]. VR exergaming has different forms of immersion. Non-immersive VR uses a small screen and a keyboard/mouse or a joystick. Semi-immersive VR uses a large screen and body parts. Immersive VR uses an HMD or a CAVE, headphones, and motion sensors. Immersive VR gives the feeling of immersion and presence. Commercial VR-based video games usually require fast movements and are not specific or task-oriented. Therefore, special video games are used for CP children’s neurorehabilitation [26,27]. Developing and validating VR games for CP is challenging. It needs collaboration among researchers, clinicians, game designers, and patients to ensure the safety, usability, efficacy, and ethics of VR. Some examples of VR games for CP are: RehabMaster™, a task-specific VR system that tracks upper limb movements with visual feedback; and RGS, a VR system that uses Kinect to monitor upper limb movements with multisensory feedback.

Home-based VR for CP can be convenient, accessible, affordable, and personalized, but also challenging due to technical, safety, supervision, and social issues. The effects of home-based vs. in-facility VR may vary by CP type and severity, VR quality and availability, therapist and caregiver support, and patient preferences and goals. More research is needed to compare different VR modalities for CP rehabilitation.

For rehabilitation programs to achieve fruitful results, sustainability and repetition are important [28]. These are difficult to maintain over the individual’s lifespan due to many barriers. Distance and cost could limit accessibility to physiotherapy centers. In addition, repetition over a long time limits children’s engagement and motivation. The use of VR-assisted exergaming in the rehabilitation of CP children has come with many advantages. Participation in these games makes the performed tasks meaningful and enjoyable for those children and therefore enhances their active engagement with the training [19,29,30]. In addition, VR technologies allow for the practice of tasks that require large physical space or cannot be performed in the real world. It can also safely simulate dangerous situations [31,32,33]. Virtual reality-based training can be provided at a low cost at home [26,27,34,35].

Several studies [20,27,29,34] were conducted to investigate the effectiveness and safety of VR-based exergaming in the rehabilitation of children with CP. We aim to provide a comprehensive overview of the current state of research on VR exergaming for CP rehabilitation. The specific objectives are: To identify and describe the existing studies that have investigated the effects of VR exergaming on motor function and participation outcomes in children with CP. In addition, we aim to identify and discuss the main gaps, challenges, and limitations in the current research on VR exergaming for CP rehabilitation. Finally, we aim to provide recommendations and suggestions for future research and practice in this field.

## 2. Methods

We conducted this secondary research following the Cochrane Handbook for systematic reviews of intervention [36]. The study was then reported according to the Preferred Reporting Items for Systematic Reviews and Meta-Analysis (PRISMA) checklist [37]. The specific details of the PRISMA checklist are in Supplementary File S1.

### 2.1. Literature Searching and Search Strategy

On 15 June 2023, we systematically searched five online databases and updated them on 5 September 2023. The following search strategy was used in all the databases: ((“Cerebral palsy OR Cerebral palsies ”) AND (“virtual reality” OR VR OR videogame OR videogames OR “video games” OR “virtual gam*” OR “augmented reality” OR “mixed reality” OR exergame* OR “Immersive virtual reality” OR IVR OR “Wii games” OR “Wii game” OR “Nintendo Wii” OR Wii OR “exergaming” OR “serious games” OR “interactive games”)). No filters or limitations were applied to the search. A search on the reference lists of the selected eligible studies was conducted manually for any additional relevant studies.

### 2.2. Eligibility Criteria

The inclusion criteria encompassed the following: (1) Population: children with CP involving the rehabilitation of children with CP; (2) Intervention: the application of VR-based exergaming on the rehabilitation; (3) Comparator: conventional rehabilitation/usual care; (4) Outcomes: Gross Motor Function Measurement (GMFM) score, PBS score, Pediatric Evaluation of Disability Inventory (PEDI) score, Canadian Occupational Performance Measure (COPM) score, WeeFunctional Independence Measure (WeeFIM) score, Melbourne Assessment of Unilateral Upper Limb Function-2 (MA-2) score, Quality of Upper Extremity Skills Test (QUEST) score, or ABILHAND-Kids test score; (5) Randomized controlled trials (RCTs) or cohort studies.

Specific studies were excluded from our analysis: (1) studies not published in English; (2) studies comprising solely of abstracts; (3) single-arm studies; and (4) studies that involved adults with cerebral palsy.

### 2.3. Selection Process

The retrieved search results were introduced to EndNote software V.20 and the duplicated results were deleted. Thereafter, we performed a screening of the titles and abstracts of the studies for initial eligibility, which means full-text screening if the paper seems to be eligible from its title and abstract. Then, the selected studies’ full texts were carefully screened for final eligibility. 

In the first phase, two reviewers independently screened the titles and abstracts of the retrieved records and excluded those that did not meet the eligibility criteria. In the second phase, the same two reviewers independently assessed the full texts of the remaining records and applied the same eligibility criteria. Any disagreement between the reviewers was resolved by discussion or consultation with a third reviewer.

### 2.4. Risk of Bias Assessment

The quality of the selected RCTs was evaluated by two investigators using Cochrane’s tool for risk of bias assessment. Bias includes (1) random sequence generation; (2) allocation concealment; (3) blinding of participants and personnel; (4) blinding of outcome assessment; (5) incomplete outcome data; (6) selective reporting; and (7) other bias [36]. Whereas the quality of cohort studies was assessed using the National Institutes of Health (NIH) tool [38]. The tool was composed of 12 questions about population and sample size justification, the research question, control definition, inclusion criteria and cases, event time, blindness, and the reporting of confounders. The authors’ opinion is classified as “good”, “fair”, or “poor” according to scores obtained during the assessment. We categorized the results of the appraisal into these three categories based on the following thresholds. Good: The study met all or most of the quality criteria and had minimal risk of bias; Fair: The study met some of the quality criteria and had a moderate risk of bias; Poor: The study met few or none of the quality criteria and had a high risk of bias. To ensure accuracy and consistency, any discrepancies during the evaluation process were resolved through discussions between the investigators or involving a third assessor.

### 2.5. Data Extraction

Summary and baseline participants’ characteristics data were extracted from each study. These data include the study design, site, and arms, the inclusion criteria, the duration of VR-based exergaming sessions, the follow-up duration, the study’s primary outcomes, and the study conclusion. In addition, these data described the studied type of CP, as well as participants’ age, gender, Gross Motor Function Classification System (GMFCS) level, Manual Ability Classification System (MACS) level, and Pediatric Balance Scale (PBS) score. Finally, we extracted the following outcomes in the data extraction step: GMFM score, PBS score, PEDI score, COPM score, WeeFIM score, MA-2 score, QUEST score, and ABILHAND-Kids test score. In addition, the specific definitions of each outcome are described below. Two reviewers independently extracted data from each study and cross-checked their results for accuracy and consistency. Any discrepancy between the reviewers was resolved by discussion or verification with the original source.

### 2.6. Study Outcomes

#### 2.6.1. Gross Motor Function Measurement (GMFM) Score

This outcome was measured through studies using two scales, the GMFM-88 scale and the GMFM-66 scale. The GMFM-88 scale consists of five dimensions: running and jumping, standing and walking, crawling and kneeling, sitting, and lying and rolling. The performance of each item was measured on a scale of five points, with higher scores indicating better capacity. The GMFM-66 scale is a subset of the GMFM-88 scale acquired via software [39,40,41].

#### 2.6.2. PBS Score

The PBS score measures the child’s ability to balance dynamically. The scale consists of 14 items; each item has a score of four points with higher scores indicating better abilities [42].

#### 2.6.3. Pediatric Evaluation of Disability Inventory (PEDI) Score

The PEDI scale assesses the child’s functioning and skills in three domains: mobility, self-care, and social functioning. Each item in the PEDI is ranked on a scale of 0–100, with a higher score indicating better performance [43].

#### 2.6.4. Canadian Occupational Performance Measure (COPM) Score

The COPM score measures the individual’s performance and satisfaction regarding daily life activities. It assesses these aspects in three domains: self-care, productivity, and leisure. Each item is self-rated on a scale of 10 points, with a higher score indicating better performance and satisfaction [44].

#### 2.6.5. WeeFunctional Independence Measure (WeeFIM) Score

The WeeFIM instrument evaluates the child’s functioning in daily life activities. Functioning in 18 items was assessed on a scale of 7 points, with a higher score indicating better performance. Six subscales are included in WeeFIM: self-care, mobility, social cognition, communication, transfer, and sphincter control [45].

#### 2.6.6. Melbourne Assessment of Unilateral Upper Limb Function-2 (MA-2) Score

This measure evaluates the function of a unilateral upper limb. Fourteen tasks are performed and recorded in video, thereafter, the movements are scored. The MA-2 score is calculated as a percentage of the maximum possible score in four subscales: range of motion, accuracy, dexterity, and fluency [46].

#### 2.6.7. Quality of Upper Extremity Skills Test (QUEST) Score

The QUEST score is a measure of the upper limb function. The QUEST scores 33 upper limb activities on a scale of two points. Finally, the score is calculated as a percentage of the maximum score. Four domains are evaluated by the QUEST: grasps, dissociated movements, protective extensions, and weight bearing [47].

#### 2.6.8. ABILHAND-Kids Test Score

ABILHAND-Kids test assesses hand disability. The test evaluates the difficulty of performing 21 bimanual activities on a scale of three points. Each activity is scored, and a higher score indicates easier performance [48].

### 2.7. Data Synthesis

We carried out a quantitative synthesis which is defined as collecting numerical data and analyzing them using statistical methods, and it aims to produce objective, empirical data that can be measured and expressed in numerical terms. Analysis was carried out using RevMan software version 5.3 in the inverse variance method. The effect estimate was calculated as a mean difference (MD) and a 95% confidence interval (CI). The heterogeneity of the results across the studies was initially evaluated by direct inspection of the forest plot. Thereafter, the results of the I-squared (I^2^) and chi-squared tests were checked. A random effect model was applied to the analysis wherever heterogeneity was detected. Then, we tried to resolve the heterogeneity by applying the “leaving one out method”. Otherwise, analysis was conducted using the fixed-effect model [49]. 

Moreover, we performed a qualitative synthesis, which is defined as collecting non-numerical data such as words, images, and sounds, and it aims to produce rich and detailed descriptions of the phenomenon being studied and to uncover new insights and meanings.

## 3. Results

### 3.1. Literature Search

**Figure 1:**
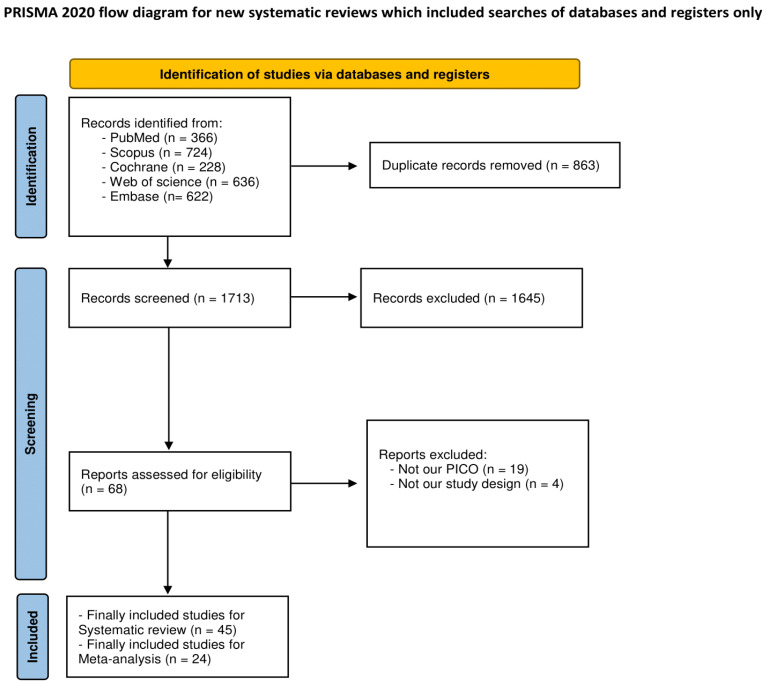
PRISMA flow diagram.

The systematic search of databases retrieved a total of 2576 studies. After removing 863 duplicates, 1713 studies underwent title and abstract screening, and 68 studies were then selected as eligible for full-text screening. Finally, 45 studies were judged eligible in this systematic review. A total of 24 studies were included in the quantitative synthesis [50,51,52,53,54,55,56,57,58,59,60,61,62,63,64,65,66,67,68,69,70,71,72,73,74], whereas 21 studies were only eligible for qualitative synthesis [75,76,77,78,79,80,81,82,83,84,85,86,87,88,89,90,91,92,93,94,95,96] (Figure 1). The manual search did not retrieve any additional relevant studies.

### 3.2. Description of the Included Studies

We included 44 RCTs and one retrospective cohort study [54] in this systematic review. The total number of enrolled CP patients was 1580 (801 for VR-based exergaming and 779 controls). The included studies were carried out in different countries: Turkey, Egypt, South Korea, Taiwan, Australia, Iran, India, the UK, Italy, Russia, Finland, Brazil, Saudi Arabia, Canada, USA, Spain, Belgium, Denmark, and the Netherlands. The duration of physiotherapy sessions ranged from 25 min to 90 min, and the frequency fluctuated between once per week and seven times a week. The studies followed the patients for a period that ranged from four to 20 weeks. Further description of the included studies’ design, conclusion, and baseline characteristics of the enrolled children is available in Table 1.

### 3.3. Quality Assessment Findings

**Figure 2:**
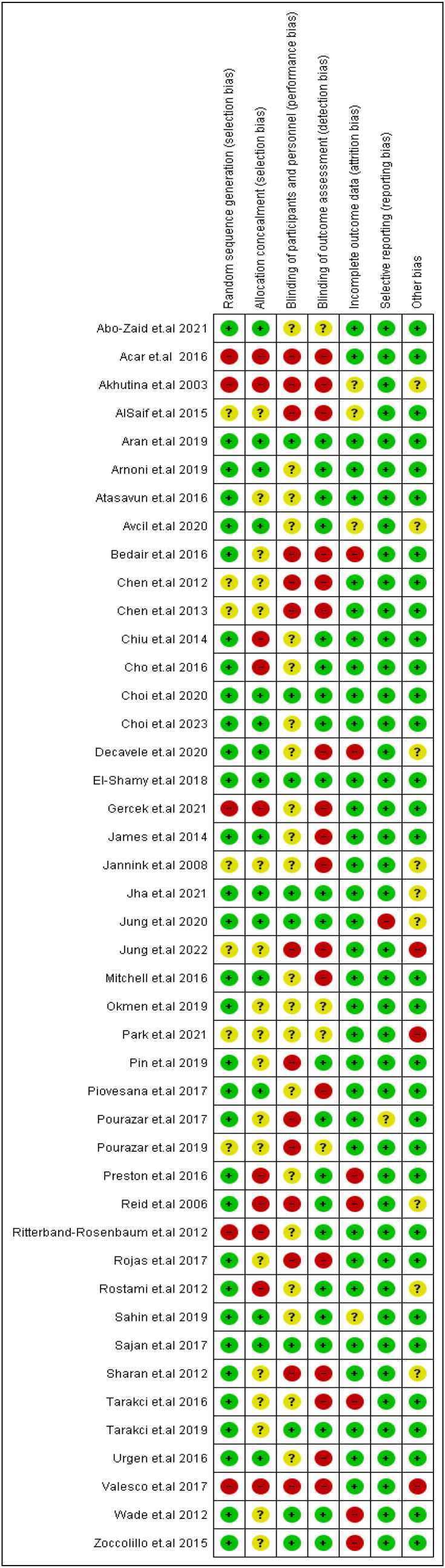
Risk of bias summary for randomized controlled trials.

The majority of the included RCTs had a low risk of bias regarding study reporting, participants’ attrition, and generating a random sequence. Allocation of the children in the study groups was concealed in less than half of the trials. Nearly half of the RCTs ensured good blinding of outcomes assessors. However, almost all the RCTs were unclear regarding the blinding of the participants and the study personnel (Figure 2). The retrospective cohort study (Chang et al., 2020) [54] had a fair quality (Supplementary File S2).

### 3.4. Results of Quantitative Syntheses

#### 3.4.1. GMFM Score

**Figure 3:**
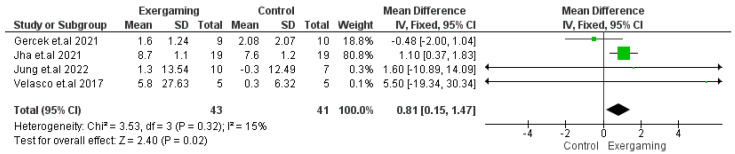
Forest plot of the analysis Gross Motor Function Measurement 88 score.

The GMFM-88 score was reported in four RCTs that enrolled 84 CP patients (43 for VR-based exergaming and 41 for controls) [51,52,53,60]. The meta-analysis revealed homogenously significant improvement in the GMFM-88 score with the use of VR-based exergaming (MD = 0.81; 95% CI [0.15, 1.47], *p*-value = 0.02), (*p*-value = 0.32, I^2^ = 15%) (Figure 3).

The GMFM-66 score was assessed in three trials, with a total of 62 enrolled children (32 for VR-based exergaming and 30 controls) [52,58,73]. The analysis showed a homogenously insignificant difference between VR-based exergaming and the control on changing the GMFM-66 score (MD = −1.26; 95% CI [−3.74, 1.22], *p*-value = 0.32), (*p*-value = 0.72, I^2^ = 0%) (Figure S1).

Four studies have reported the change in the standing dimension of the GMFM separately, with 118 enrolled children (61 for VR-based exergaming and 57 controls) [52,56,63,66]. Our analysis revealed a significant difference between the two interventions (MD = 3.15; 95% CI [0.87, 5.42], *p*-value = 0.007), and the results were homogenous (*p*-value = 0.14, I^2^ = 46%) (Figure S2).

The GMFM walking dimension was individually reported in three trials, with a sample size of 100 (52 for VR-based exergaming and 48 controls) [52,56,66]. The meta-analysis exposed homogenously significant improvement in the walking dimension with VR-based exergaming when compared to the control (MD = 1.45; 95% CI [0.48, 2.24], *p*-value = 0.003), (*p*-value = 0.98, I^2^ = 0%) (Figure S3).

#### 3.4.2. PBS Score

This meta-analysis included eight RCTs that enrolled 199 CP patients (92 for VR-based exergaming and 107 controls) [53,56,57,63,64,66,67,72]. Analysis results showed a heterogeneously insignificant difference between the studied interventions in changing the PBS score (MD = 2.83; 95% CI [−0.70, 6.35], *p*-value = 0.12), (*p*-value < 0.001, I^2^ = 98%). The detected heterogeneity could not be resolved (Figure S4).

#### 3.4.3. PEDI Score

**Figure 4:**
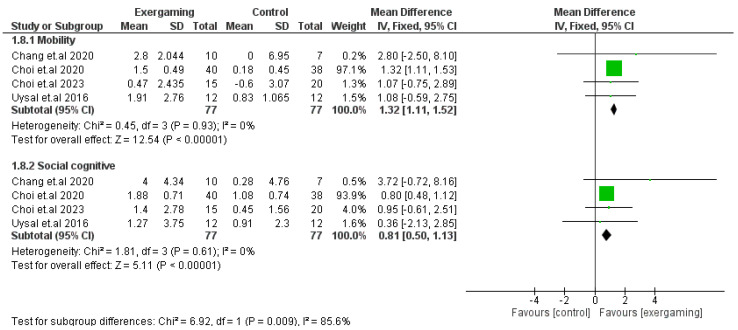
Forest plot of the analysis Pediatric Evaluation of Disability Inventory score.

The mobility and social cognitive domains were separately investigated in four RCTs that enrolled 154 CP patients (77 for each intervention) [50,54,55,67]. Our meta-analysis revealed homogenously significant improvement in the mobility and social cognitive subscales of the PEDI scale with the use of VR-based exergaming: (MD = 1.32; 95% CI [1.11, 1.52], *p*-value < 0.001) (*p*-value = 0.93, I^2^ = 0%), and (MD = 0.81; 95% CI [0.50, 1.13], *p-value <* 0.0001) (*p*-value = 0.61, I^2^ = 0%), respectively (Figure 4).

#### 3.4.4. COPM Score

**Figure 5:**
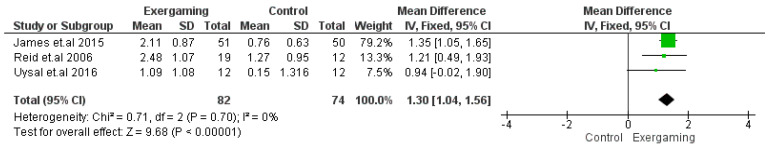
Forest plot of the analysis Canadian Occupational Performance Measure score (Performance domain).

Three trials reported the performance and satisfaction domains of COPM score, with a total of 156 enrolled children (82 for VR-based exergaming and 74 controls) [67,68,74]. The meta-analysis showed homogenously significant improvement in the performance domain with the use of VR-based exergaming (MD = 1.30; 95% CI [1.04, 1.56], *p*-value < 0.001), (*p*-value = 0.70, I^2^ = 0%) (Figure 5).

Whereas the results of satisfaction domain analysis were heterogeneous and insignificant (MD = 0.55; 95% CI [−0.74, 1.84], *p*-value = 0.41), (*p*-value < 0.0001, I^2^ = 91%). And the detected heterogeneity could not be resolved (Figure S5).

#### 3.4.5. WeeFIM Score

**Figure 6:**
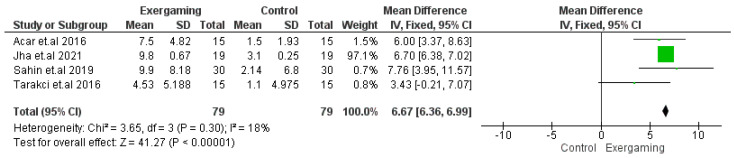
Forest plot of the analysis Wee—Functional Independence Measure total score.

The analysis of the WeeFIM total score change included four clinical trials, with 158 participants enrolled (79 for each group) [53,59,61,65]. Results of this analysis showed homogenous significant improvement in the WeeFIM score with the use of VR-based exergaming (MD = 6.67; 95% CI [6.36, 6.99], *p*-value < 0.0001) (*p*-value = 0.30, I^2^ = 18%) (Figure 6).

Two studies reported the self-care, mobility, and social cognitive domains of the WeeFIM separately, with a sample size of 90 (45 in each group). Individual analysis of the three domains revealed an insignificant variation between the two modalities: (MD = 2.14; 95% CI [−0.56, 4.85], *p*-value = 0.12), (*p*-value = 0.04, I^2^ = 76%) for the self-care domain, (MD = 0.25; 95% CI [−0.45, 0.95], *p*-value = 0.48, (*p*-value = 0.74, I^2^ = 0%) for the mobility domain, and (MD = 1.52; 95% CI [−0.61, 3.66], *p*-value = 0.16), (*p*-value = 0.19, I^2^ = 43%) for the social cognition domain (Figure S6).

#### 3.4.6. MA-2 Score

**Figure 7:**
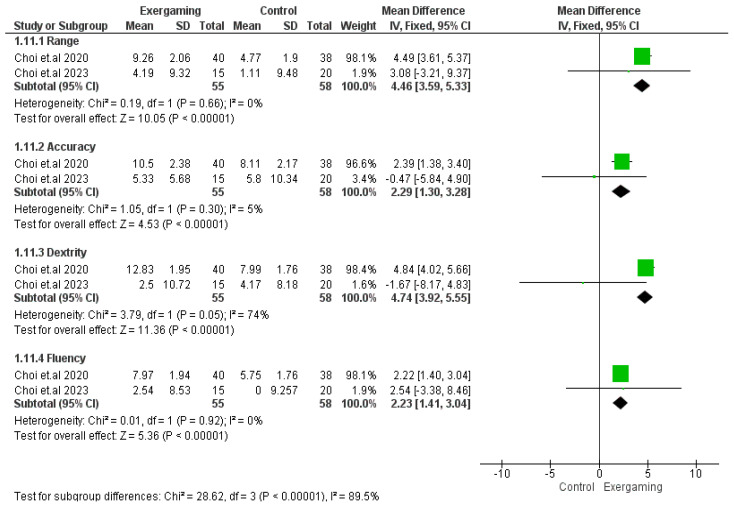
Forest plot of the analysis Melbourne Assessment of Unilateral Upper Limb Function-version 2 scale score.

The four subscales of the MA-2 score were individually reported in two trials, with 113 patients included (55 for VR-based exergaming and 58 controls) [50,55]. The meta-analyses revealed a statistically significant effect of VR-based exergaming on improving the score on the four domains: (MD = 4.46; 95% CI [3.59, 5.33], *p*-value < 0.0001), (*p*-value = 0.66, I^2^ = 0%) for the range of motion domain, (MD = 2.29; 95% CI [1.30, 3.28], *p*-value < 0.0001), (*p*-value = 0.30, I^2^ = 5%) for the accuracy domain, (MD = 4.74; 95% CI [3.92, 5.55], *p*-value < 0.0001), (*p*-value = 0.05, I^2^ = 74%) for the dexterity domain, and (MD = 2.23; 95% CI [1.41, 3.04], *p*-value < 0.0001), (*p*-value = 0.92, I^2^ = 0%) for the fluency domain (Figure 7).

#### 3.4.7. QUEST Score

Four trials were considered in the analysis of QUEST total score change, with a sample size of 97 (53 for VR-based exergaming and 44 controls) [54,64,70,74]. The analysis showed a heterogeneous insignificant variation between the two physiotherapy modalities (MD = 1.95; 95% CI [−4.82, 8.72], *p*-value = 0.57), (*p*-value = 0.04, I^2^ = 64%) (Figure S7). Chang et al., 2020 [54] was excluded in a secondary analysis that fixed heterogeneity, and the effect estimate significance remained unaffected (MD = 0.89; 95% CI [−3.51, 5.29], *p*-value = 0.69), (*p*-value = 0.20, I^2^ = 38%) (Figure S8).

Three trials reported the dissociated movements and grasp domains of the QUEST score separately, with a sample of 65 CP children (34 for VR-based exergaming and 31 controls) [54,61,64]. The two analyses showed an insignificant difference between the interventions in changing the dissociated movements domain (MD = 1.65; 95% CI [−0.61, 3.91], *p*-value = 0.15) (*p*-value = 0.62, I^2^ = 0%) and the grasps domain (MD = 4.03; 95% CI [−0.65, 8.71], *p*-value = 0.09) (*p*-value = 0.06, I^2^ = 65%) (Figures S9 and S10).

#### 3.4.8. ABILHAND-Kids Test Score

This outcome measure was reported in four RCTs that enrolled 116 CP children (58 for each intervention) [61,62,69,70]. The meta-analysis revealed a heterogeneously insignificant variation between the two studied interventions (MD = 0.29; 95% CI [−0.52, 1.09], *p*-value = 0.49) (*p*-value = 0.005, I^2^ = 77%) (Figure S11). Bedair et al., 2016 [62] was left out in a sensitivity analysis that fixed heterogeneity (*p*-value = 0.68, I^2^ = 0%), but the MD significance remained unaffected (MD = −0.05; 95% CI [−0.57, 0.46], *p*-value = 0.84) (Figure S12).

### 3.5. Qualitative Synthesis of Other Efficacy Outcomes

Park et al., 2021 reported a significantly greater improvement in the static and dynamic sitting balance (assessed via Wii Balance Board and modified functional reach test, respectively), postural swing speed and distance, as well as trunk stability with VR-based intervention when compared to the control [75]. Abo-Zaid et al., 2021 compared VR-based intervention with usual care and task-oriented therapy. Their analysis results favored task-oriented therapy over VR-based intervention and usual care regarding step length, stride length, cadence, and weight support as evaluated via the 3D motion analysis system [76]. Avcil et al., 2020 reported a similar improvement in the motor functions with the VR-based exergaming and the control. However, exergaming was more effective in improving manual dexterity as assessed via the Minnesota manual dexterity test [77].

Aran et al., 2019 reported that the VR-based exergaming was more effective in comparison with the control on improving cognitive functioning as evaluated by the dynamic occupational therapy cognitive assessment for children [78]. Ökmen et al., 2019 concluded that VR-based exergaming is more effective than the control in regards to hand function (as assessed via the bimanual fine motor function scale), functional level (assessed by the GMFCS), and mobility (evaluated by the functional mobility scale) [81]. Pourazar et al., 2019 reported better improvement in balance ability (in the anterior, posteromedial, and posterolateral directions) with VR-based exergaming when compared to the control [82]. Tarakci et al., 2019 found out that VR-based exergaming is equally beneficial as conventional physiotherapy [83].

### 3.6. Safety of the Interventions

Findings on the safety of VR-assisted exergaming were reported in 11 studies. VR-assisted appears to be safe, as no adverse events were reported in the studies [50,52,55,64,68,69,70,73,77,85].

### 3.7. GRADE Assessment

According to GRADE, all our comparisons in the different outcomes were at different levels of certainty (from very low to moderate). The causes of their downgrading were: the heterogeneity of pooled studies in each assessed outcome and the publication bias as the observational studies are more attributable to it. Moreover, the specific details and s of the publication bias are in Supplementary File S4.

## 4. Discussion

This study aimed to systematically review the evidence on the application of VR-based exergaming in rehabilitating children with CP. Our review included 45 studies (44 RCTs and one retrospective cohort study), with a total of 1580 (801 for VR-based exergaming and 779 controls). We conducted meta-analyses that revealed that VR-assisted exergaming was more effective in improving the GMFM-88 total score (*p*-value = 0.02) and the GMFM walking and standing dimensions (*p*-value < 0.05). VR-assisted exergaming modality was also more effective in improving the mobility and cognitive domains of the PEDI score (*p*-value < 0.001), the COPM performance domain (*p*-value < 0.001), and the WeeFIM total score (*p*-value < 0.001). Regarding upper limb function, VR-based exergaming was more effective (*p*-value < 0.001) in improving the four subscales of the MA-2 (accuracy, dexterity, range of motion, and fluency). VR-assisted exergaming intervention was similarly beneficial as conventional physiotherapy in improving other function assessment measures. No safety issues were observed with this proposed intervention in the included studies.

Rehabilitation by VR-assisted exergaming adds elements of fun and excitement to children’s experience with physiotherapy. These elements keep the children engaged and facilitate active participation in task-oriented training [97]. The provided direct encouraging and rewarding feedback (visual, auditory, and tactile) from the game with the aforementioned elements motivates the child to keep repeating the specific task. Eventually, the experience becomes more entertaining with higher intensity and difficulty may be increased [98,99]. Motivated active engagement, in addition to repetition of tasks and increasing difficulty, positively modulate neuroplasticity [100,101,102,103]. These facts might explain why children undergoing rehabilitation via VR-assisted exergaming have enhanced responses to rehabilitation programs. The boosted response to physiotherapy, along with the improved feasibility in terms of accessibility and cost, makes VR-assisted exergaming a promising advanced substitute to conventional physiotherapy [104,105].

The role of the Internet of Things (IoT) in VR-assisted exergaming is to enable the connection and communication between different devices and applications that are involved in the exergaming process. IoT can help to collect and process data from sensors, motion trackers, controllers, and other devices that monitor the user’s movements, performance, and feedback in the virtual environment. IoT can also help to provide remote access and support for the user, such as by allowing the therapist or caregiver to observe, guide, or join the exergaming session from a different location. IoT can enhance the value and effectiveness of VR-assisted exergaming for rehabilitation by providing more data, feedback, personalization, and social interaction [106].

Our study provides an update on the previous systematic review conducted by Fandim et al. in 2020 [107]. A total of 12 new studies are added in this update [50,51,52,53,54,55,56,57,60,75,76,77], and the number of included CP patients in the analyses is greater than before. Moreover, we conducted a separately specified meta-analysis for each function measurement scale. The results of our analyses were consistent with those reported by Fandim et al. concerning a child’s functioning and upper and lower limb mobility [106]. Heterogeneity in the results was detected in some of our meta-analyses, as in Fandim et al., 2020 [106]. This heterogeneity might emerge from the variation between the studies in the types of CP included in the study or dissimilarities in the baseline level of functioning. Moreover, the variation in the performed training tasks their difficulty and repetition, and the frequency and duration of rehabilitation sessions may contribute to the observed heterogeneity.

This systematic review and meta-analysis summarize updated evidence of the efficacy and safety of VR-assisted exergaming when applied in rehabilitating children with CP. Our study is strengthened by including a variety of studies with several exergaming techniques applied and different groups of muscles targeted. These include Bicycle exercise: This technique involves pedaling a stationary bike that is connected to a VR system that simulates different environments and scenarios. The child can choose the level of difficulty and the type of terrain they want to explore. Resistance training: This technique involves using elastic bands, weights, or machines that provide resistance to the child’s movements. The child can perform various exercises that target specific muscle groups, such as biceps curls, shoulder presses, chest presses, leg extensions, and leg curls. The resistance can be adjusted according to the child’s ability and progress. This technique can improve the strength and power of the upper and lower limb muscles. Aquatic training: This technique involves performing exercises in water that is heated to a comfortable temperature. The water provides buoyancy and resistance to the child’s movements, which can reduce pain and spasticity. The child can perform various exercises that involve moving their arms, legs, trunk, and head in different directions. This technique can improve the flexibility and range of motion of all muscle groups. Balance training: This technique involves using a VR system that provides visual and auditory feedback to the child’s balance performance. The child can stand on a platform that tilts or vibrates according to their movements. The VR system can display different scenes and challenges that require the child to maintain their balance and posture. This technique can improve the stability and coordination of the core and lower limb muscles.

This review is also strengthened by the different types of CP. However, this variation might have limited our study due to the resulting heterogeneity that could not be resolved in some of the analyses. In addition, the inconsistency in blinding the studies’ participants and personnel induces a risk of performance bias. This is explained by the inability to blind them due to the nature of the applied intervention. However, outcome assessors were properly blinded in the majority of the trials.

Many of the studies we reviewed had some methodological limitations, such as small sample sizes and lack of blinding, and most of them were preliminary tests. Therefore, we should carry out more rigorous trials on this topic and account for the potential confounders that may skew the findings. Moreover, a limitation of this systematic review is that we could not compare the effects of different types of VR (non-immersive, semi-immersive, or immersive) or different types of controls (active or passive) on the outcomes of VR exergaming for CP rehabilitation, due to the lack of information and consistency in the included studies

## 5. Conclusions

Our findings suggest that VR-assisted exergaming may have some advantages over conventional rehabilitation in improving CP children’s functioning and performance in daily life activities, upper and lower limb mobility, and cognition. VR-assisted exergaming seems to be as effective as conventional physiotherapy in the other studied function measures. With its potential efficacy, better feasibility, no reported side effects, and entertaining experience, VR-assisted exergaming may be a viable complementary approach to conventional physiotherapy in rehabilitating children with CP.

## Figures and Tables

**Table 1:** Summary of the included studies and characteristics of the enrolled patients.

N	Study ID	Study Arms, n (%)	Site	Study Design	Age, (Mean ± SD) y	Boys, n (%)	Duration of Session in Min (Frequency per Week)	Follow-up Duration (Weeks)	Arms Description, n (%)	Types of Cerebral Palsy, n (%)	MACS Level, n (%)	GMFCS Level, n (%)	Drop-Out Rate, n (%)	Inclusion Criteria	Primary Endpoints	Conclusion
1	Abo-Zaid et al., 2021	Exergaming, 20 (33.33%)	Egypt	RCT (NCT04533789)	10.3 ± 1.17	7 (35%)	60	16	Nintendo Wii	Hemiplegic	-	Between one and Two	3 (13%)	1. Unilateral CP children 2. From April 2017 to April 2019 3. Diagnosis based on careful clinical assessment by a physiotherapist	1. Swing % 2. Support % 3. Stride Length 4. Step Length 5. Cadence	“Wii Sports Training and Task-oriented Training have a significant effect on gait in children with unilateral cerebral palsy in favor of Task-oriented Training group”
					9.9 ± 0.91	6 (30%)	30 (Triple)		Neurodevelopmental control				3 (13%)			
		Control, 20 (33.33%)			10.15 ± 1.09	4 (20%)	30 (Triple)		Nintendo Wii and Neurodevelopmental control				3 (13%)			
2	Akhutina et al., 2003	Exergaming, 12 (57.14%)	Russia and Finland	RCT	7 and 14 y	7 (58%)	60 (Twice)	Four	Super Scape VRT 3-D construction packages	-	-	-	0	1. Between 7 and 14y 2. Children with Cerebral palsy 3. Given non-specific rehabilitative training	1. Difference between pre- and post-training scores	“VE-based spatial training is effective for children with complex disabilities, particularly when combined with training that remediates cognitive weaknesses”
		Control, 9 (42.86%)				5 (56%)			Two out of the nine children used wheelchairs, the others walked by themselves				0			
3	Aran et al., 2019	Exergaming, 45 (50%)	Turkey	RCT	11.18 ± 3.37	24 (53.33)	45 (Twice)	10	Exergaming and Traditional occupational therapy	Hemiplegic	a. I, 14 (31.11%) b. II, 26 (57.78%) c. III, 5 (11.11%)	a. I, 28 (62.22%) b. II, 17 (37.78%)	0	1. Children with HCP 2. Between the years 2015 and 2017 3. Aged between 7 and 12 y 4. Level I or II in GMFCS 5. Level I, II, or III of MACS	1. DOTCA-Ch scores	“Using virtual reality applications in cognitive rehabilitation was recommended to improve spatial perception, praxis, visuomotor construction and thinking operations in children with cerebral palsy”
		Control, 45 (50%)			11.06 ± 3.24	23 (51.11%)	(Twice)		Traditional occupational therapy		a. I, 12 (26.67%) b. II, 29 (64.44%) c. III, 4 (8.89%)	a. I, 31 (68.89%) b. II, 14 (31.11%)	0			
4	Arnoni et al., 2019	Exergaming, 7 (47.67%)	Brazil	Pilot (RCT)	10 ± 3.36	7 (100%)	45 (Twice)	Eight	Exergaming and Traditional occupational therapy	Mild Spastic Hemiplegic	Between Levels One and Two	a. I, 6 (85.71%) b. II, 1 (14.29%)	0	1. Between October 2013 and October 2014 2. Aged between five and 14 years 3. Levels I and II of the GMFCS 4. Have signed an informed consent	Variables of body sway: 1. Total displacement (cm) 2. Mean Velocity (cm/s)	“Intervention using an active video game is a promising tool that can improve the gross motor function of children with CP, GMFCS I-I”
		Control, 8 (53.33%)			9.39 ± 2.79	6 (75%)	-		Traditional occupational therapy			a. I, 6 (75%) b. II, 2 (25%)	0			
5	Avcil et al., 2020	Exergaming, 15 (50%)	Turkey	RCT (NCT03078998)	10.93 ± 4.09	8 (53.33%)	(Triple)	Eight	VGBT using Nintendo^®^ Wii and LMC games	1. Spastic, 12 (80%) 2. Hemiplegia, 8 (53.33%) 3. Diplegia, 4 (26.67%) 4. Dyskinetic, 3 (20%)	a. 1, 2 (13.3%) b. 2, 9 (60%) c. 3, 4 (26.7%) d. 0	a. 1, 8 (53.3%) b. 2, 4 (26.7%) c. 3, 1 (6.7%) d. 4, 2 (13.3%)	0	1. A diagnosis of Cerebral Palsy 2. The ability to cooperate with exercise or measurement	1. Minnesota Manual Dexterity Test score 2. Durouz Hand Index score	“VGBT using Nintendo^®^Wii and LMC games had slightly superior effects on manual dexterity in patients with CP while compared with NDT-based upper extremity rehabilitation. Furthermore, the effects of both treatment programs on grip strengths and functional ability were similar and beneficial”
		Control, 15 (50%)			11.07 ± 3.24	9 (60%)	-		Neuro-developmental therapy	1. Spastic, 11 (73.3%) 2. Hemiplegia, 9 (60%) 3. Diplegia, 2 (13.33%) 4. Dyskinetic, 4 (26.7%)	a. 1, 3 (20%) b. 2, 8 (53.3%) c. 3, 2 (13.3%) d. 4, 2 (13.3%)	a. 1, 3 (20%) b. 2, 2 (13.3%) c. 3, 6 (40%) d. 4, 4 (26.7%)	0			
6	Acar et al., 2016	Exergaming, 15 (50%)	Turkey	Pilot (RCT)	9.53 ± 3.04	-	45 (Twice)	Six	A neuro-developmental treatment and Nintendo Wii	Spastic	2 (SD 0.75)	a. I, 6 (40%) b. II, 9 (60%)	0	1. Aged between 6 and 15 y 2. Levels 1–3 of the MACS 3. Levels I and II of the GMFCS 4. Ability to grasp and release an object	1. QUEST total score 2. JTHFT (s) 3. WeFIM (score)	“Our results showed that neuro-developmental treatment is effective for improving hand functions in hemiplegic cerebral palsy. To provide an enjoyable, motivational, safe, and effective rehabilitation program, the Nin-tendo^®^ Wii may be used in addition to neurodevelopmental treatment”
		Control, 15 (50%)			9.73 ± 2.86	-			A neuro-developmental treatment		2 (SD 0.75)	a. I, 6 (40%) b. II, 9 (60%)	0			
7	Alsaif et al., 2015	Exergaming, 20 (50%)	Saudi Arabia	RCT	6 to 10	-	20 (Seven)	12	Nintendo Wii Fit games	Spastic diplegia	-	Level 3	0	1. Children diagnosed with CP spastic diplegia 2. Age ranged from 6–10 years old 3. Level 3 on the GMFCS 4. Lower-limb muscle power no less than grade 4	1. Balance score 2. BOTMP 5:6 3. 1-min walk test	“Using motion interactive games in-home rehabilitation is feasible for children with cerebral palsy”
		Control, 20 (50%)				-	-		No training Control				0			
8	Bedair et al., 2016	Exergaming, 20 (50%)	Egypt	RCT	7.05 ± 0.99	12 (60%)	30 (Triple)	16	VRGTP and selected physical therapy program	Spastic hemiplegic	-	-	0	1. Diagnosed as acquired spastic hemiplegic children 2. Age between 5–10 y 3. They all have the muscle tone of grade 1 or 2 4. Level of disability I–IV in the MACS	1. Visual-motor skills of PDMS-2 2. Object manipulation 3. Abilhand kids questionnaire	“Significant improvement of object manipulation, visual motor skills and upper limb functions in study group post-treatment are related to the active participation of children in the simulating environment, driven their active motivation and enhance their participation through self-competition activities. VRG can enhance active participation of children with motor deficits in the majority of upper limb activities through consideration of child personality and changing of environmental factors”
		Control, 20 (50%)			7.25 ± 0.96	11 (55%)	60 (Triple)		Selected physical therapy program				0			
9	Chang et al., 2020	Exergaming, 10 (58.83%)	South Korea	Retrospective cohort study	6.08 ±1.77	7 (70%)	VR 20 + COT 10 (Twice)	Eight	VR-based rehabilitation with RAPAEL Smart Kids and video games and COT	-	1.6 (SD 0.7)	1.1 (SD 0.32)	0	1. Children with CP 2. Age between 5-10y 3. Were selected and received two treatments	1. QUEST scores 2. PEDI domains	“Our results suggest that VR-based rehabilitation combined with COT may improve the upper extremity functions and decrease caregiver burden among children with CP”
		Control, 7 (41.17%)			4.88 ±1.15	5 (71.43%)	30 (Twice)		Conventional occupational therapy		1.42 (SD 0.78)	2.28 (SD 1.38)	0			
10	Chen et al., 2011	Exergaming, 13 (48.15%)	Taiwan	RCT	8.7 ± 2.1	9 (69.23%)	40 (Triple)	12	Home-based virtual cyclic training with games	1. Spastic diplegic, 10 (76.92%) 2. Spastic hemiplegic, 3 (23.08%)	-	a. I, 10 (76.92%) b. II, 3 (23.08%)	2 (12.5%)	1. Diagnosed CP with GMFS levels I–II 2. Age of 6–12y 3. Ability to walk independently 4. Ability to undergo a motor function and isokinetic muscle test	1. GMFM-66 scores	“Analytical findings suggest that the muscle strengthening program is more specific in enhancing bone density for children with CP than general physical activity. Thus, the proposed 12-week hVCT protocol is an effective and efficient strategy for improving lower limb aBMD in these children”
		Control, 14 (51.85%)			8.6 ± 2.2	5 (35.71%)	30–40 (Triple)		Usual and general physical activity	1. Spastic diplegic, 9 (64.29%) 2. Spastic hemiplegic, 5 (35.71%)		a. I, 11 (78.57%) b. II, 3 (21.43%)	1 (6.25%)			
11	Chen et al., 2012	Exergaming, 13 (46.43%)	Taiwan	RCT	8.7 ± 2.1	9 (69.23%)	40 (Triple)	12	Home-based virtual cyclic training with games	1. Spastic diplegic, 10 (76.92%) 2. Spastic hemiplegic, 3 (23.08%)	-	a. I, 10 (76.92%) b. II, 3 (23.08%)	0	1. Children with spastic CP 2. Diagnosis with CP with GMFCS levels I–II 3. Age 6–12 y 4. Ability to walk independently 5. Ability to undergo a motor function and isokinetic muscle test	1. BOTMP scores 2. Muscle strength (peak torque, Nm/kg)	“The protocol obtains larger gains in the knee flexor than in the knee extensor at different angular velocities. The study findings will help clinicians to provide more effective and efficient strategies for muscle strength training in children with CP”
		Control, 15 (53.57%)			8.5 ± 2.2	10 (66.67%)	-		Usual and general physical activity	1. Spastic diplegic, 10 (66.67%) 2. Spastic hemiplegic, 5 (33.33%)		a. I, 12 (80%) b. II, 3 (20%)	0			
12	Chiu et al., 2014	Exergaming, 32 (51.61%)	Taiwan and Australia	RCT	9.4 ± 1.9	15 (47%)	-	12	Home-based Wii Sports Resort training	Hemiplegic	a. I–III, 21(66) b. IV–V, 11(34)	a. I–III, 26 (81%) b. IV–V, 6 (19%)	2 (6.25%)	1. Children with spastic CP 2. Age above and below 9.5 years	1. NHPT score 2. JTHFT score	“Wii™ training did not improve coordination, strength, or hand function. Beyond the intervention, carers perceived that the children used their hands more”
		Control, 30 (48.39%)			9.5 ± 1.9	13 (43%)	-		Usual therapy only		a. I–III, 21(70) b. IV–V, 9(30)	a. I–III, 26 (87%) b. IV–V, 4 (13%)	3 (10%)			
13	Cho et al., 2016	Exergaming, 9 (50%)	South Korea	RCT	10.2 ± 3.4	-	30 (Triple)	Eight	Virtual reality with games and Tridmail training	Spastic	-	1. One, 3 (33.33%) 2. Two, 1 (11.11%) 3. Three, 5 (55.56%)	0	1. Diagnosis of spastic cerebral palsy 2. Age 4–16 y 3. Cognitive abilities enabling communication 4. GMFS level I-III	1. PBS 2. 10-min WT score 3. 2-min walk test score	“In conclusion, VRTT programs are effective for improving gait, balance, muscular strength, and gross motor function in children with CP”
		Control, 9 (50%)			9.4 ± 3.8		30 (Triple)		Tridmail training			1. One, 3 (33.33%) 2. Two, 2 (22.22%) 3. Three, 4 (44.44%)	0			
14	Choi et al., 2020	Exergaming, 40 (51.28%)	South Korea	RCT	4.33 ± 2.3	19 (47%)	30	Four	Virtual reality with games and Conventional rehabilitation	-	a. I and II, 13 (32.5%) b. III and IV, 27 (67.5%)	-	0	1. Children with CP or other acquired brain injury of at least 12 m 2. Aged 3 to 18 y 3. Children with upper-limb dysfunction 4. MACS levels I to IV and HFCS levels 4 to 7	1. MA-2 scale scores 2. ULPRS score 3. PEDI scores	“The virtual reality rehabilitation system used in this study, which consists of wearable inertial sensors and offers intensive, interactive, and repetitive motor training, is effective in children with brain injury”
		Control, 38 (48.72%)			5.67 ± 2.37	19 (50%)	60		Conventional rehabilitation		a. I and II, 16 (42.1%) b. III and IV, 22 (57.9%)		0			
15	Choi et al., 2023	Exergaming, 15 (42.86%)	Italy	RCT (KCT0003172)	8.1 ± 3.2	8 (53%)	30	Six	Training at home using the VR-enhanced program and occupational therapy	-	a. II, 8 (53%) b. III, 3 (20%) c. IV, 4 (27%)	-	2 (11.67%)	1. Children with CP or other acquired brain injury of at least 12 m 2. Aged 4–17 y 3. Children with upper-limb dysfunction 4. MACS levels I to IV and HFCS levels 4 to 7	1. MA-2 scale scores 2. ULPRS score 3. PEDI scores	“Home-based VR training though it had limited impact on improving upper limb function, could help improve social cognitive function, movement pattern, and efficiency in children with brain injury and could be an effective means of extending clinical therapy to the home”
		Control, 20 (57.14%)			7.3 ± 2.6	10 (50%)	-		Occupational therapy		a. II, 10 (53%) b. III, 5 (25%) c. IV, 5 (25%)		0			
16	Decavele et al., 2020	Exergaming, 27 (50%)	Belgium	RCT	6 to 15	-	45 (Twice)	12	Regular Physical therapy and gaming	Spastic	-	-	4 (14.8%)	1. Bilateral spastic CP 2. GMFCS level III–IV 3. Age between 6 and 15 y 4. Routinely receiving PT at an intensity of minimally twice a week	1. PBS score 2. GMFS-88 score 3. DMQ score	“A combined approach of regular PT and rehabilitation specific gaming showed significant effects on individually defined therapy goals, dynamic sitting balance, and standing exercises. However, the lack of persistent effect indicates that continuous individual goal-oriented PT with the addition of gaming is needed”
		Control, 23 (50%)				-			Regular Physical therapy				3 (13%)			
17	El-Shamy et al., 2018	Exergaming, 20 (50%)	Egypt	RCT	9.5 ± 1.2	12 (60%)	40 (Triple)	12	Wii training and usual care	Spastic Hemiplegic	a. I, 8 (40%) b. II, 9 (45%)	-	0	1. Children with CP 2. Age was 8–12 y 3. Scored I–III on MACS 4. Did not have Musculoskeletal disorders	1. Modified Ashworth scale scores 2. Spasticity scores 3. PDMS-2 scores	“Wii training plus usual care decreases spasticity and increases grip strength and hand function in children with hemiplegic cerebral palsy”
		Control, 20 (50%)			9.8 ± 1.4	14 (70%)	60 (Triple)		Usual care only		a. I, 7 (35%) b. II, 9 (45%)		0			
18	Gercek et al., 2021	Exergaming, 9 (47.37%)	Turkey	RCT	8.22 ± 1.71	7 (77.78%)	60 (Triple)	12	Correct golf swing during the virtual game	Spastic hemiplegic	a. I, 3 (33.33%) b. II, 6 (66.67%)	-	0	1. Children had CP at GMFCS level one or two 2. Age 6–12 y 3. With sufficient hand function to hold the golf club	1. GMFM-88 score 2. 6 min Walk(m) score	“Both virtual and traditional golf training applied for 12 weeks on children with unilateral cerebral palsy improved lower extremity functions and physical performance. The use of virtual and traditional training as complementary applications to reduce motor problems in children with cerebral palsy could enhance the sustainability of this type of training because of its edutainment features. Virtual golf has an important advantage over traditional golf in that (a) the latter can be expensive and inaccessible for people with disabilities, and (b) making virtual golf a safer activity”
		Control, 10 (52.63%)			8.5 ± 2.5	7 (70%)			Correct golf swing		a. I, 3 (30%) b. II, 7 (70%)		0			
19	Gatica-Rojas et al., 2017	Exergaming, 16 (50%)	Australia	RCT	10.2 ± 3.1	10 (62.50%)	30 (Triple)	10	Nintendo Wii balance and SPT	Hemiplegia, 4 (44.44%)	-	a. I, 3 (37.50%) b. ii, 5 (62.5%)	0	1. CP type SHE and SDI 2. Level I or II of GMFCS 3. Males and females aged between 7 and 14y	1. COP for open and closed eyes 2. Mean velocities (cm/s)	“A systematic exercise program like Wii therapy using the Nintendo Wii balance board device can be considered to improve the standing balance in patients with CP, specifically in the SHE type. This program is easy to transfer to physiotherapists and rehabilitation centers”
		Control, 16 (50%)			11.2 ± 3.6	9 (56.25%)	40 (Triple)		Standard physiotherapy	Hemiplegia, 5 (55.55%)		a. I, 3 (33.33%) b. II, 6 (66.66%)	0			
20	Okmin et al., 2019	Exergaming, 21 (51.22%)	Turkey	RCT	8.8 ± 2.5	14 (66.67%)	60 (Triple)	Four	VR and (Neurophysiological, conventional, and occupational therapy)	1. Spastic, 20 (95.5%) 2. Dyskinetic athetoid, 1 (4.8%)	-	-	0	1. Age between 5 and 15 y 2. Being able to cooperate and being motivated 3. Having sitting balance and normal UE passive	1. Bimanual fine motor function score 2. GMFCS score	“Our study results indicate that VR therapy is a useful treatment method that can be used in the rehabilitation of CP with improved motor function. The addition of this method to conventional rehabilitation techniques may have a significant impact on treatment success”
		Control, 20 (48.78%)			8.2 ± 1.8	14 (70%)			Neurophysiological, conventional, and occupational therapy	1. Spastic, 19 (95%) 3. Mixed, 1 (5%)			0			
21	James et al., 2015	Exergaming, 47 (51.09%)	Australia	RCT	11.67 ± 2.33	26 (51%)	30 (Hexable)	20	Mitii (a web camera using green tracking bands worn on the hands, knee, or head)and occupational therapy	Hemiplegia, 28 (55%)	a. I, 11 (21.6%) b. II, 39 (76.5%) c. III, 1 (2%)	1. I, 20 (39.2%) 2. II, 31 (60.8%)	4 (7.8%)	1. Children with spastic type UCP 2. MACS 1 and 3 3. GMFCS 1 and 2 4. Aged 8 to 18y 5. Internet access at home	1. COPM score 2. MUUL score 3. Test of Visual Perceptual Skills score	“ Mitii delivers individualized, web-based therapy at home and has the potential to increase the therapy dose. Mitii can be considered as an option to enhance occupational performance and visual perception for children with UCP”
		Control, 45 (48.91%)			11.83 ± 2.42	25 (50%)			Occupational therapy	Hemiplegia, 20 (40%)	a. I, 13 (26%) b. II, 37 (74%) c. III, 0	1. I, 25 (50%) 2. II, 25 (50%)	5 (10%)			
22	Jannink et al., 2008	Exergaming, 5 (50%)	Netherlands	Pilot(RCT)	12.32 ± 3.18	5 (100%)	30 (Twice)	Six	Low-Cost Video Game and physiotherapy	1. Spastic tetraplegia, 4 (80%) 2. Spastic diplegia, 1 (20%)	Two, Three, and Four	One and Four	0	1. Age between 7 and 16 y 2. Have a diagnosis of CP 3. Can understand the Dutch language 4. Can stretch and bend the shoulder and elbow of their affected arm	1. Melbourne score assessment	“In conclusion, it can be said that the Eye Toy is a motivational training tool for the training of children with CP and has the potential to improve upper extremity function”
		Control, 5 (50%)			11.88 ± 3.47	4 (80%)			Physiotherapy	1. Spastic tetraplegia, 3 (60%) 2. Spastic Hemiplegia, 1 (20%) 3. Spastic diplegia, 1 (20%)	Two, Three, and Four	One, Three, and Four	0			
23	Jha et al., 2021	Exergaming, 19 (50%)	India	RCT	8.94 ± 1.92	14 (74%)	60 (Pentable)	Six	Combined virtual gaming and physiotherapy session	Spastic	a. 1, 12 (63%) b. 2, 7 (33%)	a. 2, 17 (89%) b. 3, 2 (11%)	1 (5.3%)	1. Children with bilateral spastic CP 2. Aged 6–12y 3. With an ability to understand simple verbal instructions 4. GMFCS level II–III 5. MACS level I–II	1. PBS score 2. GMFM-88 score 3. WeFIM score	“Combined virtual reality gaming and physiotherapy are not superior over physiotherapy alone in improving the gross motor performance and daily functioning among children with bilateral spastic cerebral palsy. Virtual gaming, along with physiotherapy, appears to be beneficial in their balance capacity, warranting further trials to investigate the same in children with GMFCS level III”
		Control, 19 (50%)			8.72 ± 1.68	9 (47%)	30 (Pentable)		Physiotherapy session		a. 1, 10 (53%) b. 2, 9 (47%)	a. 2, 16 (84%) b. 3, 3 (16%)	1 (5.3%)			
24	Jung et al., 2020	Exergaming, 5 (50%)	South Korea	Pilot (RCT)	12.8 ± 1.6	3 (60%)	80 (Triple)	Six	Kinect Video Game Training and Conventional therapy	Spastic diplegia	a. 1, 2 (40%) b. 2, 3 (60%)	a. 1, 2 (40%) b. 2, 3 (60%)	0	1. Adolescents with CP 2. Aged 11–17 y 3. Levels I and II of the GFMCS 4. Levels I and II of the MACS	1. Selective control assessment score 2. PBS score	“KVG training might be an effective intervention for the rehabilitation of adolescents with spastic diplegia CP”
		Control, 5 (50%)			12 ± 2.53	2 (40%)	40 (Triple)		Conventional therapy		a. 1, 3 (60%) b. 2, 2 (40%)	a. 1, 3 (60%) b. 2, 2 (40%)	0			
25	Jung et al., 2022	Exergaming, 10 (58.83%)	South Korea	RCT	9.34 ± 2.11	7 (70%)	30 (Twice)	Eight	HRS sessions with VR training and conventional physiotherapy	Spastic	-	a. I. 6 (60%) b. II. 1 (10%) c. III. 1 (10%) d. IV. 2 (20%)	0	1. Diagnosed as spastic CP 2. Aged between 5 and 18 y 3. Level I-IV of the GFMCS 4. Able to sit astride on a saddle	1. GMFM-88 score 2. GMFM-66 score	“HRS with VR may be an effective adjunctive therapeutic approach for the rehabilitation of children with CP”
		Control, 7 (41.17%)			9.1 ± 2.41	4 (57.14%)			Conventional physiotherapy			a. I. 4 (57.14%) b. II. 1 (14.29%) c. III. 0 d. IV. 2 (28.57%)	0			
26	Piovesana et al., 2016	Exergaming, 51 (50.5%)	Australia	RCT (ACTRN12611001174976)	11.63 ± 2.30	26 (51%)	30 (Hexable)	20	Mitii (a web camera using green tracking bands worn on the hands, knee, or head)and occupational therapy	Hemiplegia, 28 (55%)	a. I, 11 (21.6%) b. II, 39 (76.5%) c. III, 1 (2%)	a. I, 20 (39.2%) b. II, 31 (60.8%)	4 (7.8%)	1. Children with UCP 2. GMFCS-E&R I or II 3. MACS level I–II and III 4. Aged 8–18y 5. Received upper-limb or lower-limb surgery	1. BRIEF scores 2. Executive functioning scores	“In a large RCT, MitiiTM did not lead to significant improvements on measures of EF or parent ratings of EF performance in children with UCP”
		Control, 50 (49.5%)			11.86 ± 2.45	25 (50%)			Occupational therapy	Hemiplegia, 20 (40%)	a. I, 13 (26%) b. II, 37 (74%) c. III, 0	a. I, 25(50%) b. II, 25(50%)	6 (12%)			
27	Pourazar et al., 2017	Exergaming, 15 (50%)	Iran	RCT	10.9 ± 0.87	15 (100%)	25 (Triple)	Four	Xbox 360 Kinect as a therapeutic device for VR intervention	Spastic hemoplegic	a. I, 9 (60%) b. II, 1 (6.67%)	-	0	1. Between the ages of 7 and 12 y 2. Scores range from 1 to 3 in GFMCS 3. MACS level I–II 4. Able to walk without an assistive device	1. Simple Reaction Time 2. Discriminative Reaction Time	“This paper proposes VR as a promising tool into the rehabilitation process for improving reaction time in children with cerebral palsy”
		Control, 15 (50%)			11.5 ± 0.52	15 (100%)			Common therapy programs by the school during the intervention		a. I, 8 (53.33%) b. II, 2 (13.33%)		0			
28	Pourazar et al., 2019	Exergaming, 10 (50%)	Iran	Pilot (RCT)	9.2 ± 1.4	-	(Triple)	Six	Xbox 360 Kinect as a therapeutic device for video game-based training	Spastic hemiplegic	a. I, 7 (70%) b. II, 3 (30%)	a. I, 5 (50%) b. II, 2 (20%) c. III, 3 (30%)	0	1. Girls ranging from 7 to 12 y 2. Spastic Hemiplegic Cerebral Palsy 3. MACS level I–II 4. Scores range from 1 to 3 in GMFCS	1. One-way covariance test scores	“This paper proposes that video game-based training can successfully guide children with cerebral palsy to improve their balance ability. This virtual system is therefore an interesting tool in the therapies related to children with cerebral palsy”
		Control, 10 (50%)			9.6 ± 1.5				Typical physical activity under parental supervision at home		a. I, 9 (90%) b. II, 1 (10%)	a. I, 6 (60%) b. II, 3 (30%) c. III, 1 (10%)	0			
29	Rosenbaum et al., 2012	Exergaming, 20 (50%)	Denmark	RCT	11.1 ± 2.1	14 (70%)	30 (Triple)	20	Internet-based home training system ‘Move It to Improve It’ (MiTii)	Spastic	-	I–II and III	0	1. Diagnosed as spastic CP 2. Level I-IV of the GFMCS 3. Able to sit astride on a saddle	1. Kinematics of the movements	“These findings suggest that sense of agency may be altered and that training in the sense of agency may help to increase the outcome of training programs in children with CP”
		Control, 20 (50%)			12 ± 2.6	12 (60%)			Continued their regular daily activities				0			
30	Rostami et al., 2012	Exergaming, 8 (25%)	Iran	Pilot(RCT)	7.73 ± 0.795	3 (37.5%)	90 (Triple)	Four	VR with games	Spastic hemiparesis	-	-	0	1. Age range 6 y 2 mo to 11 y 8 mo 2. With spastic hemiparetic CP 3. At least 20◦wrist and 10◦active finger extension from full flexion	1. Quality of movement score 2. Speed and dexterity score	“Modified constraint-induced movement therapy in a virtual environment could be a promising rehabilitation procedure to enhance the benefits of both virtual reality and constraint-induced therapy techniques”
		mCIMT, 8 (25%)			8.29 ± 1.2	4 (50%)			Modified Constraint-Induced Movement Therapy				0			
		Exergaming and mCIMT, 8 (25%)			8.5 ± 1.01	4 (50%)			VR and mCIMT				0			
		Control, 8 (25%)			8.46 ± 1.61	3 (37.5%)			Regular routines of treatment programs that had been prescribed before enrolment in the study				0			
31	Mitchell et al., 2016	Exergaming, 51 (50.5%)	Australia	RCT	11.25 ± 2.33	26 (51%)	30 (Hexable)	20	Physical activity games were interspersed with upper-limb and visual perceptual games	-	a. I, 11 (22%) b. II, 39 (76%) c. III, 1 (2%)	a. I, 21 (41%) b. II, 30 (59%)	4 (7.8%)	1. Children and adolescents aged 8 to 17 y 2. With unilateral CP 3. Levels one and two in GFMCS 4. Levels one and three in MACS	1. Physical activity capacity scores 2. Physical activity performance scores	“Training was effective at increasing functional strength and walking endurance in independently ambulant children with unilateral CP. This did not translate into improvements in activity performance”
		Control, 50 (49.5%)			11.33 ± 2.5	26 (52%)			Occupational physiotherapy		a. I, 13 (26%) b. II, 37 (74%) c. III, 0	a. I, 25 (50%) b. II, 25 (50%)	7 (13.72%)			
32	Park et al., 2021	Exergaming, 10 (50%)	South Korea	RCT	14.3 ± 4.2	3 (30%)	60 (Twice)	Four	Balance training in the sitting position using a VR training program	1. Diplegia, 5 (50%) 2. Quadriplegia, 5 (50%)	-	-	0	1. Children aged 6–18 y 2. Diagnosed with spastic CP 3. Had spastic quadriplegia and 10 had spastic paraplegias 4. Children with no vision and hearing problems 5. With an appropriate cognitive level	1. Total path length (cm) 2. Velocity (cm/s)	“Posture control training in the sitting position using a VR training program was found to be more effective in improving the sitting balance and trunk stability of children with CP”
		Control, 10 (50%)			14.1 ± 4.3	4 (40%)	40 (Twice)		Arm reach training in the sitting position	1. Diplegia, 5 (50%) 2. Quadriplegia, 5 (50%)			0			
33	Pin et al., 2019	Exergaming, 9 (50%)	UK	Pilot (RCT) (NCT02975804)	8.92 (2.25)	5 (56%)	20 (Pentable)	12	Interactive computer play training in sitting four times per a week and (muscle strength physiotherapy program)	Bilateral spastic CP	-	a. III, 8 (88.88%) b. IV, 1 (11.11%)	0	1. Children with moderate cerebral palsy 2. GMFCS level III or IV 3. Were aged between 6 and 14 y 4. Were able to follow instructions	1. GMFM-66 score	“The intervention protocol of a six-week interactive computer play training was feasible and safe for children with moderate cerebral palsy in special school settings. Future studies with larger sample sizes or using single-subject designs are recommended”
		Control, 9 (50%)			9.59 (1.87)	6 (67%)	-		Muscle strength physiotherapy program			a. III, 8 (88.88%) b. IV, 1 (11.11%)	0			
34	Preston et al., 2015	Exergaming, 8 (53.33%)	UK	RCT (ISRCTN26206379)	9.41 ± 2.41	4 (50%)	30	12	The computer-assisted arm rehabilitation gaming technology	Spastic	a. II, 2 (25%) b. III, 3 (37.5%) c. IV, 3 (37.5%)	-	0	1. Children aged five to 12 y 2. with a diagnosis of cerebral palsy who were to receive botulinum toxin 3. MACS levels one and two 4. Arm capability sufficient to manipulate the handle of the robotic arm and vision	1. ABILHAND-kids function scores 2. Canadian Occupational Performance Measure scores	“This study suggests that computer-assisted arm rehabilitation gaming does not benefit arm function, but a Type II error cannot be ruled out”
		Control, 7 (46.67%)			8.58 ± 2.58	5 (71.43%)			Receive a matching visit for delivery or collection of the device		a. II, 1 (14.29%) b. III, 2 (28.57%) c. IV, 4 (57.14%)		0			
35	Reid et al., 2006	Exergaming, 19 (61.29%)	Canada	Pilot(RCT)	9.75 ± 1.55	12 (63.16%)	90 (Once)	Eight	Sat on a bench or in a wheelchair viewing a large TV screen in a well-demarcated area	-	-	-	0	1. Children with CP 2. Aged between 8 and 10 y	1. COPM-P score 2. COPM-S score 3. QUEST scores	“These findings will be discussed to suggest that VR remains a viable rehabilitation tool and future research needs to be done where strategies for control group attention are devised as well as its use in reaction therapy”
		Control, 12 (28.71%)			9.23 ± 1.19	8 (66.67%)			The standard of care which is occupational therapy or physical therapy				0			
36	Sahin et al., 2019	Exergaming, 30 (50%)	Turkey	RCT	10.5 ± 3.62	20 (66.67%)	45 (Twice)	Eight	Box, Wii, Superkick, and Jet Run	Spastic	-	-	0	1. Aged between 7 and 16 y 2. Having a>24 score of MMSE for children 3. MACS levels one and two 4. Having been classified in levels I–II of MACS 5. levels I–III of the GMFCS 6. Able to follow and accept verbal instructions	1. BOTMP-SF scores 2. WeeFIM scores	“The Kinect-based VR intervention approach is important to improving motor functions and independence in daily activities of children with USCP”
		Control, 30 (50%)			10.06 ± 3.24	17 (56.67%)			Traditional occupational therapy				0			
37	Sajan et al., 2016	Exergaming, 10 (50%)	India	RCT (CTRI/2011/11/002137)	10.6 ± 3.78	5 (50%)	45 (Hexable)	Three	Wii-based interactive video games and conventional therapy	1. Spastic, 7 (70%) 2. Triplegia, 1 (10%) 3. Quadriplegia, 2 (20%)	-	a. I, 1 (10%) b. II, 2 (20%) c. III, 6 (60%) d. IV, 1 (10%) e. VI, 0	1 (10%)	1. Children with CP 2. Children aged 5–20 y 3. With sufficient balance to play Wii games in the sitting or standing position 4. Children with a history of serious health problems	1. Sway velocity for eyes (mm/s) 2. QUEST scores 3. TVPS scores	“Wii-based IVG may be offered as an effective supplement to conventional therapy in the rehabilitation of children with CP”
		Control, 10 (50%)			12.4 ± 4.93	6 (60%)	-		A goal-directed, comprehensive rehabilitation program that involved a team of Various specialties	1. Spastic,5 (50%) 2. Diplegia, 4 (40%) 3. Quadriplegia, 1 (10%)		a. I, 0 b. II, 1 (10%) c. III, 7 (70%) d. IV, 2 (20%) e. VI, 0	1 (10%)			
38	Sharan et al., 2012	Exergaming, 14 (48.28%)	India	Pilot (RCT)	8.88 ± 3.23	-	-	Three	(Nintendo Wii Sports and Wii Fit) and CR	-	-	-	0	1. Diagnosed as spastic CP 2. Level I–IV of the GFMCS 3. Able to sit astride on a saddle	1. PBS score 2. MACS score	“To the author’s best knowledge, this is the first study on using VR-based therapy for the postoperative rehabilitation of children with CP which needs further elaboration with larger sample size”
		Control, 15 (51.72%)			10.38 ± 4.41				Conventional rehabilitation modalities				0			
39	Tarakci et al., 2016	Exergaming, 15 (50%)	Turkey	Pilot RCT	10.46 ± 2.69	10 (66.67%)	50 (Twice)	12	(Wii-Fit balance-based video game training) and NDT	1. Hemiplegic, 7 (47%) 2. Diplegic, 5 (33%) 3. Dyskinetic, 3 (20%)	-	2 (Range 1–2)	0	1. Age 5–18 y 2. GMFCS level 1, level 2, or level 3 3. No history of epilepsy and no botulinum toxin A 4. Between February 2011 and February 2013	1. PBS score 2. MACS score	“Wii-fit balance-based video games are better at improving both static and performance-related balance parameters when combined with NDT treatment in children with mild CP”
		Control, 15 (50%)			10.53 ± 2.79	9 (60%)			Neuro-developmental treatment	1. Hemiplegic, 7 (47%) 2. Diplegic, 7 (47%) 3. Dyskinetic, 1 (7%)		2 (Range 1–3)	0			
40	Tarakci et al., 2019	Exergaming, 15 (50%)	Turkey	RCT	10.93 ± 4.09	8 (53.33%)	60 (Triple)	Eight	Leap Motion Controller-based training	1. Spastic, 12(80) 2. Dyskinetic, 3(20)	-	-	4 (21%)	1. Between April 2016 and July 2017 2. All participants have signed informed consent forms 3. Aged between 5 to 17 y	1. Durouz Hand Index score 2. JTHFT score 3. CHAQ score 4. NHPT score	“This study has quantitatively shown that LMCBT should be used as an effective alternative treatment option in children and adolescents with physical disabilities”
		Control, 15 (50%)			11.06 ± 3.23	9 (60%)			Conventional Rehabilitation	1. Spastic, 11 (73.3%) 2. Dyskinetic, 4 (26.7%)			4 (21%)			
41	Urgen et al., 2016	Exergaming, 15 (50%)	Turkey	RCT	11.07 ± 2.37	7 (46.6%)	45 (Twice)	Nine	Nintendo ^®^Wii-Fit and conventional rehabilitation	Spastic Hemiplegic	-	I and II	0	1. Children with hemiplegic spastic CP 2. Age from 7 to 14y 3. GMFCS level II 4. To attend the routine rehabilitation program	1. GMFM scores 2. Soft floor eyes open and closed balance reactions 3. Single Leg Standing and Tandem Standing 4. TUG(s)	“Nintendo^®^Wii-Fit training may affect advanced motor skills and improve the balance of children with spastic hemiplegic CP with physiotherapy”
		Control, 15 (50%)			11.33 ± 2.19	7 (46.6%)	-		Routine Physiotherapy and Rehabilitation Program				0			
42	Uysal et al., 2016	Exergaming, 12 (50%)	Turkey	Pilot RCT	9.13 ± 2.57	8 (66.7%)	30 (Twice)	Four	The NW gaming console with the Wii Sports Games was used for training and PT	Spastic	a. I, 6 (50%) b. II, 2 (16.7%) c. III, 4 (33.3%)	a. I, 9 (75%) b. II, 3 (25%)	0	1. Children diagnosed with CP 2. Between the ages of 6 and 14 y 3. Level I or II on the GMFCS 4. Level I, II, or III on the MACS	1. PEDI scores 2. PBS score 3. COPM score	“NW contributed to the implementation of occupational performance, daily living activities, and functional balance. We recommend that NW could be used in the rehabilitation program to engage in play-based activities with fun”
		Control, 12 (50%)			10.11 ± 2.62	2 (16.7%)	-		Regular physiotherapy		a. I, 6 (50%) b. II, 3 (25%) c. III, 3 (25%)	a. I, 10 (83.3%) b. II, 2 (16.7%)	0			
43	Velasco et al., 2017	Exergaming, 5 (50%)	Spain	RCT	4.8 ± 3	-	30 (Twice)	16	Serious videogames and (Traditional physical and occupational therapy)	-	-	-	0	1. Males and females, aged 4–21 y 2. Diagnosed CP and cervical hypotonia or difficulties with head control 3. Cognitive capacity and behavior appropriate to understand the tasks	1. GMFM-88 scores 2. VAS score 3. GAS score	“Physical therapy that combines serious games with traditional rehabilitation could allow children with CP to achieve larger function improvements in the trunk and cervical regions. However, given the limited scope of this trial (n = 10) additional studies are needed to corroborate this hypothesis”
		Control, 5 (50%)			11.2 ± 3.8		-		Traditional physical and occupational therapy				0			
44	Wade et al., 2012	Exergaming, 6 (46.15%)	UK	RCT	Mean 9.1	-	(Triple)	Six	Computer and equipment with seat and platform(VR)	-	-	IV or V	0	1. Young people aged 5 to 16 y 2. level IV or V on the GMFCS 3. At level three or higher on the Chailey scale of sitting ability	1. Chailey level of box-sitting ability scores	“The study provides evidence to suggest that a meaningful and engaging therapeutic activity, such as using computer games controlled by leaning the upper body, can help to improve sitting ability in children with neuromotor dysfunction. Further work is required to understand fully what effects such activities have on the various components of sitting ability”
		Control, 7 (53.85%)					-		No intervention				0			
45	Zoccolillo et al., 2015	Exergaming, 11 (50%)	Italy	RCT	6.89 ± 1.91	-	90 (Triple)	Eight	Xbox with Kinect device and Conventional therapy	-	-	-	1 (9%)	1. Clinical diagnosis of CP 2. Age between 4 and 14 y 3. GMFCS level 1 to 4	1. ABILHAND kids function scores	“VGT resulted effective in improving the motor functions of upper limb extremities in children with cerebral palsy, conceivably for the increased quantity of limb movements, but failed in performing the manual abilities for performing activities of daily living which benefited more from CT”
		Control, 11 (50%)					60 (Twice)		Conventional therapy				3 (27.3%)			

## Data Availability

Data are available upon reasonable request. Data are available from the corresponding author upon reasonable request.

## Supplementary Materials

The following supporting information can be downloaded at: https://www.mdpi.com/article/10.3390/jcm12227091/s1, Supplementary File S1: PRISMA 2020 Checklist. Supplementary File S2: Quality assessment of the retrospective cohort study (Chang et al. 2020). Supplementary File S3: Figure S.1: Forest plot of the analysis Gross Motor Function Measurement score-66; Figure S2: Forest plot of the analysis Gross Motor Function Measurement-Standing dimension score; Figure S3: Forest plot of the analysis Gross Motor Function Measurement-Walking dimension score; Figure S4: Forest plot of the analysis of the Pediatric Balance Scale score; Figure S5: Forest plot of the analysis of Canadian Occupational Performance Measure-Satisfaction domain score; Figure S6: Forest plot of the analysis Wee – Functional Independence Measure score; Figure S7: Forest plot of the analysis Quality of Upper Extremity Skills Test total score; Figure S8: Forest plot of the analysis Quality of Upper Extremity Skills Test total score after leaving one out; Figure S9: Forest plot of the analysis Quality of Upper Extremity Skills Test-Dissociated movements domain score; Figure S10: Forest plot of the analysis Quality of Upper Extremity Skills Test-Grasps domain score; Figure S11: Forest plot of the analysis of ABILHAND kids’ test scores; Figure S12: Forest plot of the analysis of ABILHAND kid’s test scores after leaving one out. Supplementary File S4 detailing VR compared to control for Cerebral Palsy.
