# Supplementary material for: Application of Virtual Reality-Assisted Exergaming on the Rehabilitation of Children with Cerebral Palsy: A Systematic Review and Meta-Analysis
Source: J Clin Med. 2023 Nov 14;12(22):7091. doi: 10.3390/jcm12227091 (PMC10672287; doi:10.3390/jcm12227091)
Supplement: Supplementary file 1 [file jcm-12-07091-s001.zip › jcm-2661647-supplementary/Suplementary files Updated/Supplementary file 2.pdf]

Table S1: Quality assessment of the retrospective cohort study (Chang et al. 2020)

ID	NIH Quality Assessment Tool for Observational Cohort and Cross-Sectional Studies															Quality rating: good (11-14) or fair (7.5-10.5) or poor (0-7), Yes = 1 // No = 0.5 // NR & NA & CD = 0
	1. Was the research question or objective in this paper clearly stated?	2. Were eligibility/selection criteria for the study population prespecified and clearly described?	3. Were the participants in the study representative of those who would be eligible for the test/service/intervention in the general or clinical population of interest?	4. Were all eligible participants that met the prespecified entry criteria enrolled?	5. Was the sample size sufficiently large to provide confidence in the findings?	6. For the analyses in this paper, were the exposure(s) of interest measured before the outcome(s) being measured?	7. Was the time frame sufficient so that one could reasonably expect to see an association between exposure and outcome if it existed?	8. For exposures that can vary in amount or level, did the study examine different levels of the exposure as related to the outcome (eg, categories of exposure, or exposure measured as a continuous variable)?	9. Were the exposure measures (independent variables) clearly defined, valid, reliable, and implemented consistently across all study participants?	10. Was the exposure(s) assessed more than once over time?	11. Were the outcome measures prespecified, clearly defined, valid, reliable, and assessed consistently across all study participants?	12. Were the people assessing the outcomes blinded to the participants' exposures/interventions?	13. Was the loss to follow-up after baseline 20% or less? Were those lost to follow-up accounted for in the analysis?	14. Were key potential confounding variables measured and adjusted statistically for their impact on the relationship between exposure(s) and outcome(s)?	Total scores	
	Yes / No / Not reported (NR) or cannot determine (CD) or not applicable (NA)	Yes / No / Not reported (NR) or cannot determine (CD) or not applicable (NA)	Yes / No / Not reported (NR) or cannot determine (CD) or not applicable (NA)	Yes / No / Not reported (NR) or cannot determine (CD) or not applicable (NA)	Yes / No / Not reported (NR) or cannot determine (CD) or not applicable (NA)	Yes / No / Not reported (NR) or cannot determine (CD) or not applicable (NA)	Yes / No / Not reported (NR) or cannot determine (CD) or not applicable (NA)	Yes / No / Not reported (NR) or cannot determine (CD) or not applicable (NA)	Yes / No / Not reported (NR) or cannot determine (CD) or not applicable (NA)	Yes / No / Not reported (NR) or cannot determine (CD) or not applicable (NA)	Yes / No / Not reported (NR) or cannot determine (CD) or not applicable (NA)	Yes / No / Not reported (NR) or cannot determine (CD) or not applicable (NA)	Yes / No / Not reported (NR) or cannot determine (CD) or not applicable (NA)	Yes / No / Not reported (NR) or cannot determine (CD) or not applicable (NA)		
**Chang et.al 2020 [1]**	Yes	NR	Yes	NR	No	Yes	No	NA	Yes	NR	Yes	NR	Yes	Yes	**8**	**Fair**

References:

1. Chang HJ, Ku KH, Park YS, Park JG, Cho ES, Seo JS, et al., editors. Effects of virtual reality-based rehabilitation on upper extremity function among children with cerebral palsy2020 2020: MDPI.
