# Supplementary material for: Application of Virtual Reality-Assisted Exergaming on the Rehabilitation of Children with Cerebral Palsy: A Systematic Review and Meta-Analysis
Source: J Clin Med. 2023 Nov 14;12(22):7091. doi: 10.3390/jcm12227091 (PMC10672287; doi:10.3390/jcm12227091)
Supplement: Supplementary file 1 [file jcm-12-07091-s001.zip › jcm-2661647-supplementary/Suplementary files Updated/Supplementary File 3.pdf]

Supplementary File 3: Supplementary Figures 1 to 12

Study or Subgroup	Exergaming Mean	Exergaming SD	Exergaming Total	Control Mean	Control SD	Control Total	Weight	Mean Difference IV, Fixed, 95% CI
Chen et.al 2011	2	6.61	13	3.5	4.31	14	34.1%	-1.50 [-5.74, 2.74]
Jung et.al 2022	1.9	11.24	10	-0.8	9.63	7	6.2%	2.70 [-7.27, 12.67]
Pin et.al 2019	0.51	2.73	9	2.04	4.08	9	59.7%	-1.53 [-4.74, 1.68]
**Total (95% CI)**			**32**			**30**	**100.0%**	**-1.26 [-3.74, 1.22]**

Heterogeneity: Chi² = 0.65, df = 2 (P = 0.72); I² = 0%
Test for overall effect: Z = 1.00 (P = 0.32)

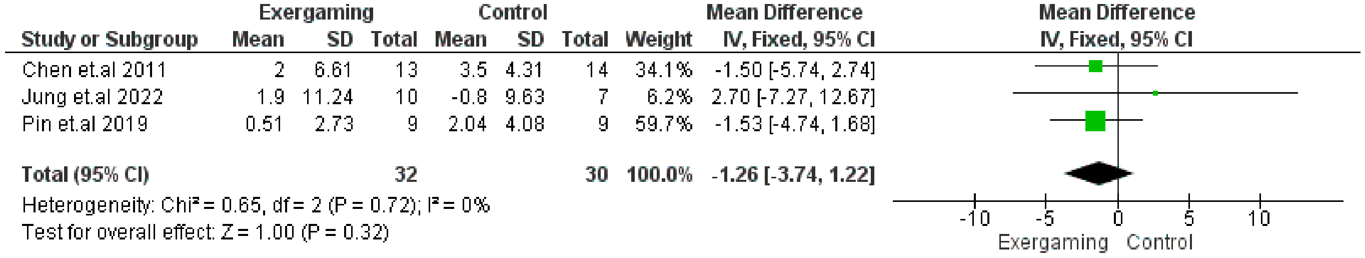

Figure S1: Forest plot of the analysis Gross Motor Function Measurement score-66

Study or Subgroup	Exergaming Mean	Exergaming SD	Exergaming Total	Control Mean	Control SD	Control Total	Weight	Mean Difference IV, Random, 95% CI
Cho et.al 2016	9.1	3.23	9	3.2	3.85	9	25.8%	5.90 [2.62, 9.18]
Decavele et.al 2020	3.8	5.94	27	0.2	5.9	23	25.8%	3.60 [0.31, 6.89]
Jung et.al 2022	1.8	8.75	10	-0.3	1.41	10	13.1%	2.10 [-3.39, 7.59]
Urgen et.al 2016	5.64	2.48	15	4.45	3.86	15	35.3%	1.19 [-1.13, 3.51]
**Total (95% CI)**			**61**			**57**	**100.0%**	**3.15 [0.87, 5.42]**

Heterogeneity: Tau² = 2.42; Chi² = 5.55, df = 3 (P = 0.14); I² = 46%
Test for overall effect: Z = 2.71 (P = 0.007)

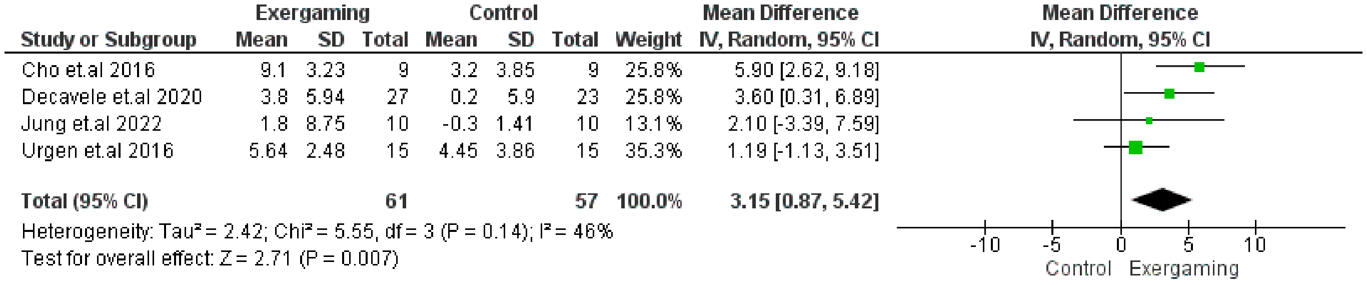

Figure S2: Forest plot of the analysis Gross Motor Function Measurement-Standing dimension score

Study or Subgroup	Exergaming Mean	Exergaming SD	Exergaming Total	Control Mean	Control SD	Control Total	Weight	Mean Difference IV, Fixed, 95% CI
Decavele et.al 2020	1	2.02	27	-0.5	1.966	23	76.7%	1.50 [0.39, 2.61]
Jung et.al 2022	0.7	1.1	10	-0.3	16.06	10	0.9%	1.00 [-8.98, 10.98]
Urgen et.al 2016	2.69	2.29	15	1.39	3.347	15	22.3%	1.30 [-0.75, 3.35]
**Total (95% CI)**			**52**			**48**	**100.0%**	**1.45 [0.48, 2.42]**

Heterogeneity: Chi² = 0.04, df = 2 (P = 0.98); I² = 0%
Test for overall effect: Z = 2.93 (P = 0.003)

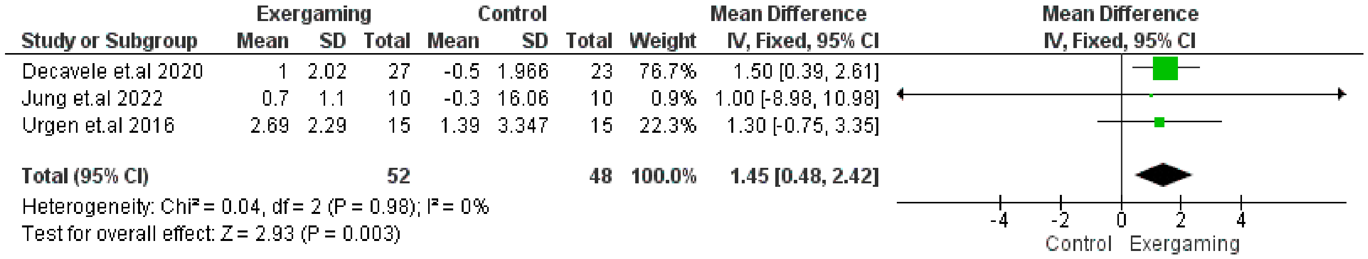

Figure S3: Forest plot of the analysis Gross Motor Function Measurement-Walking dimension score

Study or Subgroup	Exergaming Mean	Exergaming SD	Exergaming Total	Control Mean	Control SD	Control Total	Weight	Mean Difference IV, Random, 95% CI
Uysal et.al 2016	1.67	1.08	12	0.25	0.85	12	14.0%	1.42 [0.64, 2.20]
Urgen et.al 2016	3.73	1.69	15	0.8	1.597	15	13.9%	2.93 [1.75, 4.11]
Sharan et.al 2012	9.43	6.85	14	9.67	6.8	15	11.0%	-0.24 [-5.21, 4.73]
Sajan et.al 2016	3	2.98	9	4.56	3.17	9	12.9%	-1.56 [-4.40, 1.28]
Jung et.al 2020	16.8	1.44	5	2.4	0.01	5	13.8%	14.40 [13.14, 15.66]
Jha et.al 2021	5	0.84	19	3.7	1.02	19	14.0%	1.30 [0.71, 1.89]
Decavele et.al 2020	1.3	7.43	9	-0.2	1.966	23	11.1%	1.50 [-3.42, 6.42]
Cho et.al 2016	3.3	7.07	9	2.1	7.43	9	9.3%	1.20 [-5.50, 7.90]
**Total (95% CI)**			**92**			**107**	**100.0%**	**2.83 [-0.70, 6.35]**

Heterogeneity: Tau² = 22.98; Chi² = 372.03, df = 7 (P < 0.00001); I² = 98%
Test for overall effect: Z = 1.57 (P = 0.12)

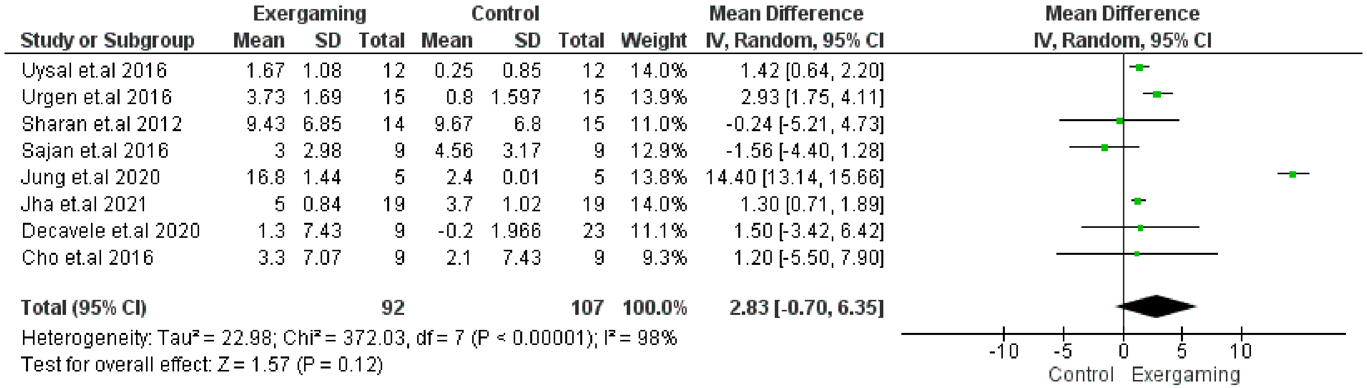

Figure S4: Forest plot of the analysis of the Pediatric Balance Scale score.

Study or Subgroup	Exergaming Mean	Exergaming SD	Exergaming Total	Control Mean	Control SD	Control Total	Weight	Mean Difference IV, Random, 95% CI
James et.al 2015	2.08	1	51	0.58	0.81	50	36.3%	1.50 [1.15, 1.85]
Reid et.al 2006	2.69	0.79	19	1.82	1.2	12	32.9%	0.87 [0.10, 1.64]
Uysal et.al 2016	0.53	0.84	12	1.45	1.49	12	30.8%	-0.92 [-1.89, 0.05]
**Total (95% CI)**			**82**			**74**	**100.0%**	**0.55 [-0.74, 1.84]**

Heterogeneity: Tau² = 1.16; Chi² = 21.83, df = 2 (P < 0.0001); I² = 91%
Test for overall effect: Z = 0.83 (P = 0.41)

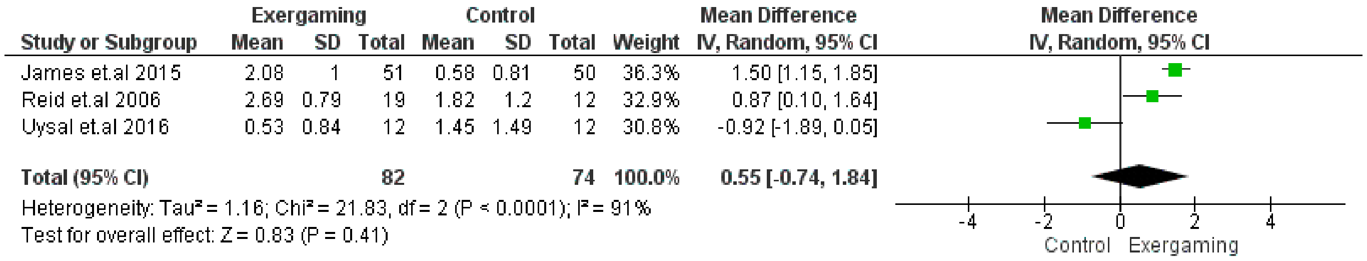

Figure S5: Forest plot of the analysis of Canadian Occupational Performance Measure-Satisfaction domain score

Study or Subgroup	Exergaming Mean	Exergaming SD	Exergaming Total	Control Mean	Control SD	Control Total	Weight	Mean Difference IV, Random, 95% CI
**1.19.1 Self care**								
Sahin et.al 2019	4.13	3.9	30	0.63	3.3	30	50.7%	3.50 [1.67, 5.33]
Tarakci et.al 2016	1.14	2.2	15	0.4	3.15	15	49.3%	0.74 [-1.20, 2.68]
**Subtotal (95% CI)**			**45**			**45**	**100.0%**	**2.14 [-0.56, 4.85]**
Heterogeneity: Tau² = 2.88; Chi² = 4.11, df = 1 (P = 0.04); I² = 76%								
Test for overall effect: Z = 1.55 (P = 0.12)								
**1.19.2 Mobility**								
Sahin et.al 2019	0.64	2.04	30	0.5	1.7	30	53.8%	0.14 [-0.81, 1.09]
Tarakci et.al 2016	0.4	1.53	15	0.02	1.33	15	46.2%	0.38 [-0.65, 1.41]
**Subtotal (95% CI)**			**45**			**45**	**100.0%**	**0.25 [-0.45, 0.95]**
Heterogeneity: Tau² = 0.00; Chi² = 0.11, df = 1 (P = 0.74); I² = 0%								
Test for overall effect: Z = 0.71 (P = 0.48)								
**1.19.3 Soscial cognition**								
Sahin et.al 2019	1.2	1.8	30	0.3	1.7	30	75.3%	0.90 [0.01, 1.79]
Tarakci et.al 2016	4.53	5.188	15	1.1	4.975	15	24.7%	3.43 [-0.21, 7.07]
**Subtotal (95% CI)**			**45**			**45**	**100.0%**	**1.52 [-0.61, 3.66]**
Heterogeneity: Tau² = 1.38; Chi² = 1.75, df = 1 (P = 0.19); I² = 43%								
Test for overall effect: Z = 1.40 (P = 0.16)								

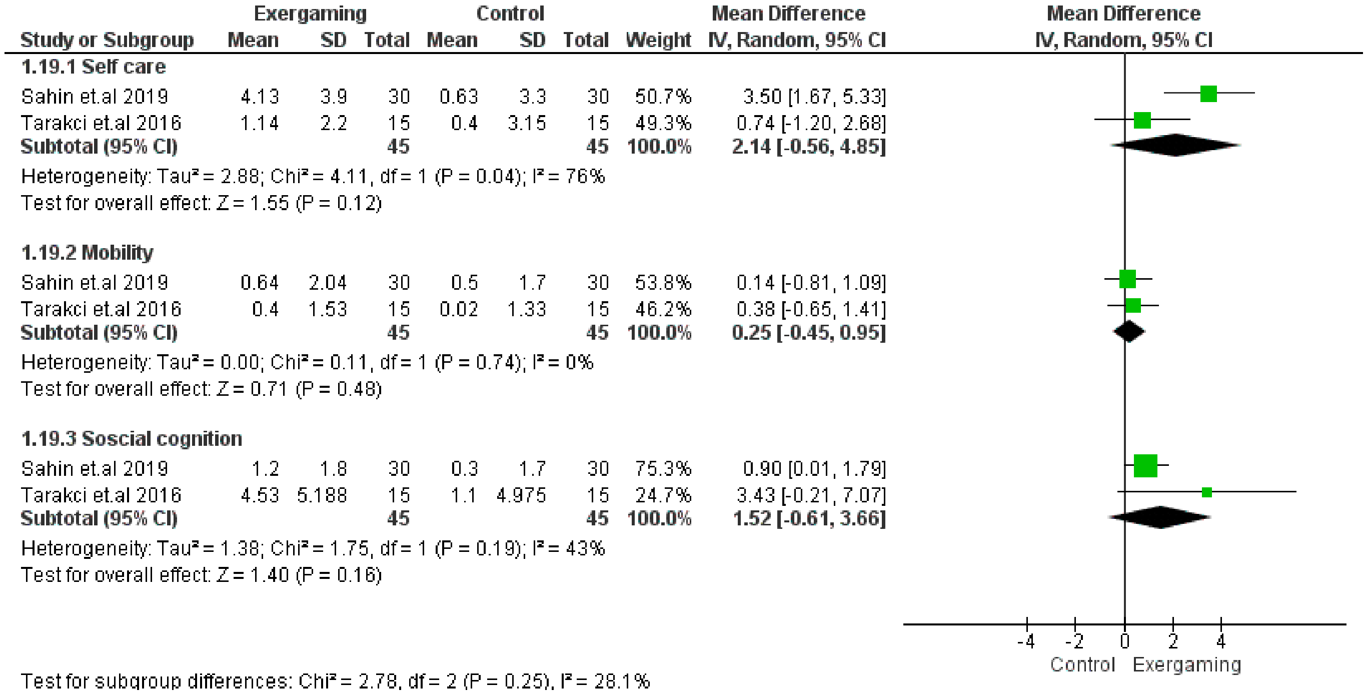

Test for subgroup differences: Chi² = 2.78, df = 2 (P = 0.25), I² = 28.1%

Figure S6: Forest plot of the analysis Wee – Functional Independence Measure score

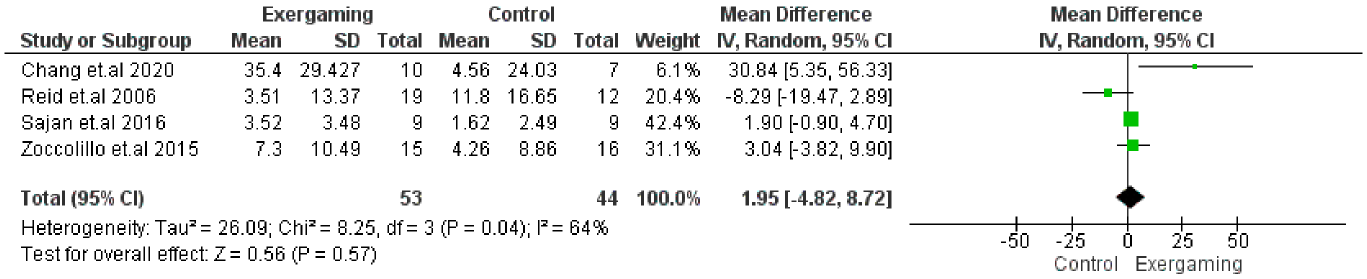

Figure S7: Forest plot of the analysis Quality of Upper Extremity Skills Test total score

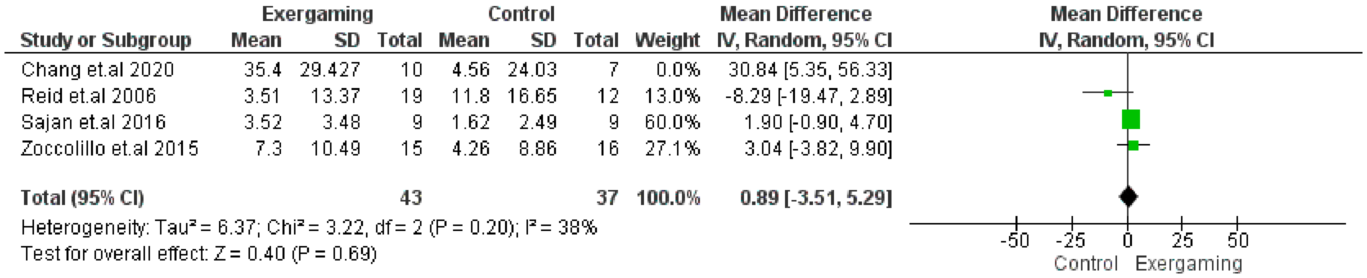

Figure S8: Forest plot of the analysis Quality of Upper Extremity Skills Test total score after leaving one out.

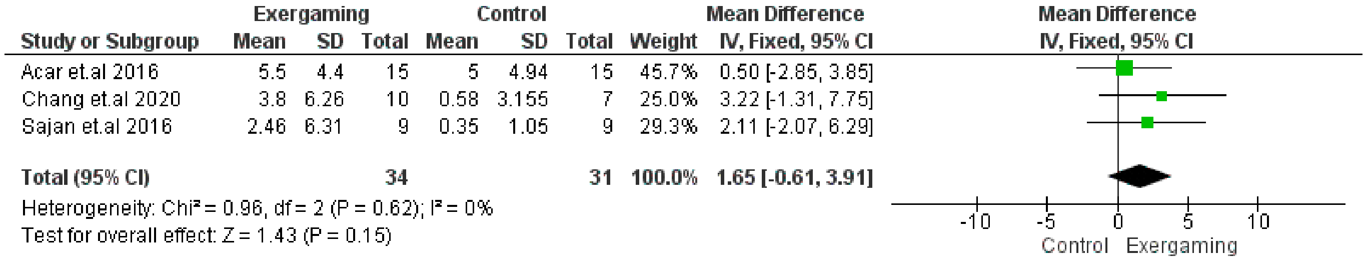

Figure S9: Forest plot of the analysis Quality of Upper Extremity Skills Test-Dissociated movements domain score.

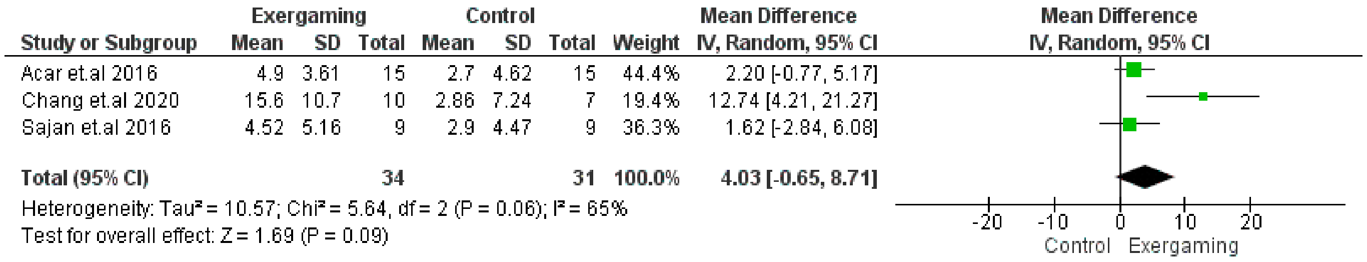

Figure S10: Forest plot of the analysis Quality of Upper Extremity Skills Test-Grasps domain score.

Study or Subgroup	Exergaming Mean	Exergaming SD	Exergaming Total	Control Mean	Control SD	Control Total	Weight	Mean Difference IV, Random, 95% CI
Acar et.al 2016	0.73	1.16	15	0.6	0.63	15	30.1%	0.13 [-0.54, 0.80]
Bedair et.al 2016	2.14	0.581	20	1.1	0.49	20	35.7%	1.04 [0.71, 1.37]
Preston et.al 2015	-0.676	0.7125	8	-0.385	0.94	7	26.6%	-0.29 [-1.14, 0.56]
Zoccolillo et.al 2015	1	2.99	15	1.63	4.36	16	7.5%	-0.63 [-3.25, 1.99]
**Total (95% CI)**			**58**			**58**	**100.0%**	**0.29 [-0.52, 1.09]**

Heterogeneity: $Tau^2 = 0.44$; $Chi^2 = 12.95$, df = 3 (P = 0.005); $I^2 = 77\%$
Test for overall effect: Z = 0.70 (P = 0.49)

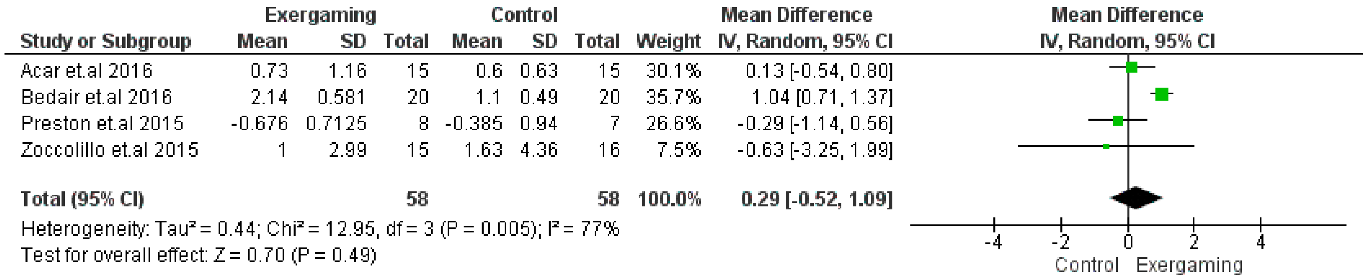

Figure S11: Forest plot of the analysis of ABILHAND kids' test scores

Study or Subgroup	Exergaming Mean	Exergaming SD	Exergaming Total	Control Mean	Control SD	Control Total	Weight	Mean Difference IV, Random, 95% CI
Acar et.al 2016	0.73	1.16	15	0.6	0.63	15	59.6%	0.13 [-0.54, 0.80]
Bedair et.al 2016	2.14	0.581	20	1.1	0.49	20	0.0%	1.04 [0.71, 1.37]
Preston et.al 2015	-0.676	0.7125	8	-0.385	0.94	7	36.5%	-0.29 [-1.14, 0.56]
Zoccolillo et.al 2015	1	2.99	15	1.63	4.36	16	3.9%	-0.63 [-3.25, 1.99]
**Total (95% CI)**			**38**			**38**	**100.0%**	**-0.05 [-0.57, 0.46]**

Heterogeneity: $Tau^2 = 0.00$; $Chi^2 = 0.77$, df = 2 (P = 0.68); $I^2 = 0\%$
Test for overall effect: Z = 0.20 (P = 0.84)

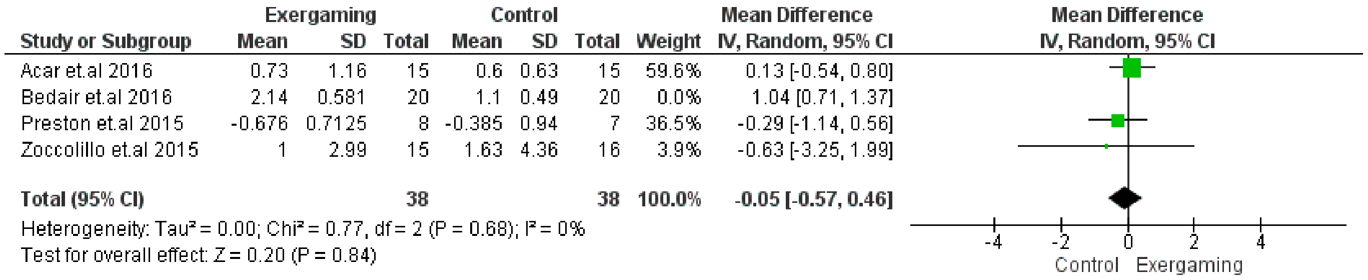

Figure S12: Forest plot of the analysis of ABILHAND kid's test scores after leaving one out.
