# Supplementary material for: Application of Virtual Reality-Assisted Exergaming on the Rehabilitation of Children with Cerebral Palsy: A Systematic Review and Meta-Analysis
Source: J Clin Med. 2023 Nov 14;12(22):7091. doi: 10.3390/jcm12227091 (PMC10672287; doi:10.3390/jcm12227091)
Supplement: Supplementary file 1 [file jcm-12-07091-s001.zip › jcm-2661647-supplementary/Suplementary files Updated/Supplementary file 4.pdf]

Author(s):

Question: VR compared to control for Cerebral palsy

Setting:

Bibliography:

Certainty assessment							№ of patients		Effect		Certainty	Importance
№ of studies	Study design	Risk of bias	Inconsistency	Indirectness	Imprecision	Other considerations	VR	control	Relative (95% CI)	Absolute (95% CI)		
**Gross Motor Function Measurement score-66**												
3	randomised trials	serious	serious	not serious	not serious	none	32	30	-	MD **1.26 lower** (3.74 lower to 1.22 higher)	⨁⨁◯◯ Low	NOT IMPORTANT
**Gross Motor Function Measurement score-88**												
4	randomised trials	serious	not serious	not serious	not serious	publication bias strongly suspected	43	41	-	MD **0.81 higher** (0.15 higher to 1.47 higher)	⨁⨁◯◯ Low	IMPORTANT
**Gross Motor Function Measurement (Dimension D=Standing)**												
4	randomised trials	serious	not serious	not serious	not serious	publication bias strongly suspected	61	57	-	MD **3.15 higher** (0.87 higher to 5.42 higher)	⨁⨁◯◯ Low	IMPORTANT
**Gross Motor Function Measurement (Dimension E=Walking)**												
3	randomised trials	serious	not serious	not serious	not serious	none	52	48	-	MD **1.45 higher** (0.48 higher to 2.42 higher)	⨁⨁⨁◯ Moderate	IMPORTANT
**Pediatric Evaluation of Disability Inventory score (Mobility subgroup)**												
4	randomised trials	serious	not serious	not serious	not serious	publication bias strongly suspected	77	77	-	MD **1.32 higher** (1.11 higher to 1.52 higher)	⨁⨁◯◯ Low	IMPORTANT
**Pediatric Evaluation of Disability Inventory score (Social cognitive subgroup)**												
4	randomised trials	serious	not serious	not serious	not serious	publication bias strongly suspected	77	77	-	MD **0.81 higher** (0.5 higher to 1.13 higher)	⨁⨁◯◯ Low	IMPORTANT
**Wee – Functional Independence Measure (Total Score)**												

Certainty assessment							№ of patients		Effect		Certainty	Importance
№ of studies	Study design	Risk of bias	Inconsistency	Indirectness	Imprecision	Other considerations	VR	control	Relative (95% CI)	Absolute (95% CI)		
4	randomised trials	not serious	not serious	not serious	not serious	none	79	79	-	MD **1.28 lower** (6.77 lower to 4.2 higher)	⊕⊕⊕⊕ High	CRITICAL
**The Melbourne Assessment of Unilateral Upper Limb Function-version 2 scale score**												
2	randomised trials	not serious	serious	not serious	not serious	publication bias strongly suspected	55	58	-	MD **0.69 higher** (0.36 higher to 1.35 higher)	⊕⊕◯◯ Low	IMPORTANT
**Pediatric Balance Scale score**												
8	randomised trials	very serious	not serious	not serious	not serious	none	92	107	-	MD **2.83 higher** (0.7 lower to 6.35 higher)	⊕⊕◯◯ Low	NOT IMPORTANT
**Canadian Occupational Performance Measure score (Satisfaction)**												
3	randomised trials	very serious	not serious	not serious	not serious	publication bias strongly suspected	82	74	-	MD **0.55 higher** (0.74 lower to 1.84 higher)	⊕◯◯◯ Very low	NOT IMPORTANT
**Wee – Functional Independence Measure**												
2	randomised trials	serious	not serious	not serious	serious	none	45	45	-	MD **2.14 higher** (0.56 lower to 4.95 higher)	⊕⊕◯◯ Low	
**Quality of Upper Extremity Skills Test (Total score)**												
4	observational studies	very serious	serious	not serious	not serious	none	53	44	-	MD **1.95 higher** (4.82 lower to 8.72 higher)	⊕◯◯◯ Very low	NOT IMPORTANT
**QUEST score (Dissociated movements)**												
3									-	**0** (0 to 0)	-	
**QUEST score (Grasps)**												
3	observational studies	very serious	serious	not serious	not serious	none	34	31	-	MD **1.65 higher** (0.61 lower to 3.91 higher)	⊕◯◯◯ Very low	NOT IMPORTANT
**ABILHAND kids test score**												

Certainty assessment							№ of patients		Effect		Certainty	Importance
№ of studies	Study design	Risk of bias	Inconsistency	Indirectness	Imprecision	Other considerations	VR	control	Relative (95% CI)	Absolute (95% CI)		
4	observational studies	very serious	not serious	not serious	not serious	none	34	31	-	MD **4.03 higher** (0.65 lower to 8.71 higher)	⨁◯◯◯ Very low	NOT IMPORTANT

CI: confidence interval; **MD:** mean difference
